# Spatial and Temporal Analysis of Gene Expression during Growth and Fusion of the Mouse Facial Prominences

**DOI:** 10.1371/journal.pone.0008066

**Published:** 2009-12-16

**Authors:** Weiguo Feng, Sonia M. Leach, Hannah Tipney, Tzulip Phang, Mark Geraci, Richard A. Spritz, Lawrence E. Hunter, Trevor Williams

**Affiliations:** 1 Department of Craniofacial Biology, University of Colorado Denver, Aurora, Colorado, United States of America; 2 Department of Cell and Developmental Biology, University of Colorado Denver, Aurora, Colorado, United States of America; 3 Department of Pharmacology, University of Colorado Denver, Aurora, Colorado, United States of America; 4 Department of Medicine, University of Colorado Denver, Aurora, Colorado, United States of America; 5 Human Medical Genetics Program, University of Colorado Denver, Aurora, Colorado, United States of America; Brunel University, United Kingdom

## Abstract

Orofacial malformations resulting from genetic and/or environmental causes are frequent human birth defects yet their etiology is often unclear because of insufficient information concerning the molecular, cellular and morphogenetic processes responsible for normal facial development. We have, therefore, derived a comprehensive expression dataset for mouse orofacial development, interrogating three distinct regions – the mandibular, maxillary and frontonasal prominences. To capture the dynamic changes in the transcriptome during face formation, we sampled five time points between E10.5–E12.5, spanning the developmental period from establishment of the prominences to their fusion to form the mature facial platform. Seven independent biological replicates were used for each sample ensuring robustness and quality of the dataset. Here, we provide a general overview of the dataset, characterizing aspects of gene expression changes at both the spatial and temporal level. Considerable coordinate regulation occurs across the three prominences during this period of facial growth and morphogenesis, with a switch from expression of genes involved in cell proliferation to those associated with differentiation. An accompanying shift in the expression of polycomb and trithorax genes presumably maintains appropriate patterns of gene expression in precursor or differentiated cells, respectively. Superimposed on the many coordinated changes are prominence-specific differences in the expression of genes encoding transcription factors, extracellular matrix components, and signaling molecules. Thus, the elaboration of each prominence will be driven by particular combinations of transcription factors coupled with specific cell:cell and cell:matrix interactions. The dataset also reveals several prominence-specific genes not previously associated with orofacial development, a subset of which we externally validate. Several of these latter genes are components of bidirectional transcription units that likely share *cis*-acting sequences with well-characterized genes. Overall, our studies provide a valuable resource for probing orofacial development and a robust dataset for bioinformatic analysis of spatial and temporal gene expression changes during embryogenesis.

## Introduction

The face provides an important template for integrating major sensory inputs from the mouth, nose, eyes, and ears that are then relayed to the adjacent brain. The development and evolution of the face and jaws has been a major driving force in the expansion of vertebrate lineages over recent geological times. Paired jaws have served as critical components for adaptive radiation, and variation in jaw design between species has generated many different vertebrate facial morphologies [1]. Moreover, for humans and many other species, the face provides a fundamental aspect of a person's individuality, acts as a major component of sexual selection, and serves as the vessel through which our emotions are relayed to others.

Although some growth of the face occurs post-natally, the basic facial pattern is generated during embryogenesis by a complex set of tissue interactions and morphogenetic processes (for reviews see [2], [3], [4]). Growth and patterning of the face relies on several small buds of tissue, the facial prominences, which surround the primitive mouth. These prominences consist of swellings of mesenchyme that are encased in an overlying epithelium. The mammalian upper jaw is derived from six main prominences: two central medial nasal processes, flanked by paired lateral nasal and maxillary prominences. The lateral and medial nasal prominences are components of the frontonasal mass, while the maxillary prominence is derived from tissue rostral to the first branchial arch. The lower jaw originates from a pair of mandibular prominences, each derived from the first branchial arch. Beginning around E10 of mouse development, the prominences undergo rapid growth and morphogenesis. By E11.5 the paired medial nasal prominences are in close apposition in the midline, and these structures also abut the maxillary prominences on each side of the developing face. By E12.5 the nasal and maxillary prominences fuse to form a continuous shelf at the front of the face - the primary palate. The formation of the secondary palate from outgrowths of the maxillary prominences is a separate developmental process that occurs later, between E12-E15.5 of mouse embryogenesis [5].

The interaction of at least five tissues is vital for the formation of the face: neural crest cells (NCCs), paraxial mesoderm, the neural tube, the foregut endoderm, and the facial ectoderm [2], [4]. The first two tissues form most of the facial skeleton, connective tissue, and muscle. In combination with the latter three tissues they also supply growth factors and signaling molecules needed for the appropriate growth and patterning of the facial skeleton. NCCs from the hindbrain region form ganglia and skeletal derivatives associated with the branchial arches, including the mandible and hyoid bones. NCCs from the midbrain and forebrain regions form a major component of the frontonasal mesenchyme. These forebrain and midbrain NCC populations will form the cartilage, bone and connective tissue of the face, while the musculature will be derived from the contribution of paraxial mesoderm to the facial mesenchyme.

Concomitant with these morphogenetic processes, a number of growth, differentiation, and patterning events also occur. Some events, such as the development of the cartilage and bone of the craniofacial skeleton, are shared among all the facial prominences. In other instances, individual prominences are associated with specific developmental processes, and this is reflected by patterns of differential gene expression that give the prominences their unique identities. Thus, the mandibular and maxillary prominences give rise to dentition, the frontonasal prominence has a unique role in olfaction, and the mandibular prominence in taste.

Reflecting the complexity of facial development, orofacial defects are among the most frequent human congenital malformations [6], [7], [8]. Cleft lip with or without cleft palate (CL/P) occurs in approximately 1 per 1000 live births in North America. Lateral clefts in CL/P are caused by failure of the lateral nasal and maxillary prominences to fuse and may be either unilateral or bilateral. CL/P can also occur in the facial midline due to a failure in the fusion of the two medial nasal prominences at the ventral midline. Cleft secondary palate (cleft palate; CP) results from aberrant formation, morphogenesis, and fusion of the maxillary prominence-derived palatal shelves at a slightly later stage of orofacial development. In addition to clefts, a number of other rare birth defects involve abnormal growth of the underlying craniofacial skeleton [9].

Orofacial clefts and other craniofacial malformations have clear environmental and genetic causes. However, insufficient information exists concerning the mechanisms of orofacial development to detect or prevent the majority of these defects pre-natally. In this context, the identification and characterization of the genes expressed during embryonic facial development will provide valuable insight into the molecular and cellular interactions governing this process. Here, using the mouse as a model system, we have employed gene expression profiling to study the pathways involved in face formation. We focused on the cellular and molecular changes occurring in the developing mouse face between E10.5–E12.5 during which the facial prominences undergo dynamic growth, morphogenesis and fusion to form the basic platform of the face. Determining the molecular mechanisms driving face development during this period provides a critical framework for understanding the genetic and environmental causes of human orofacial clefting as well as other facial dysmorphologies.

## Materials and Methods

### Study Design

All animal experiments were performed in accordance with protocols approved by the University of Colorado Denver (UCD) Animal Care and Usage Committee. Our analysis focused upon Theiler stages (TS) 17–20.3, which are equivalent to E10.5–E12.5 of mouse embryonic development. This time-span is the most relevant to the development of human orofacial clefts, and also marks the period in which initial differentiation and morphogenesis of the craniofacial skeleton occurs [3], [10], [11]. The earliest time point (E10.5) corresponds to when all three facial prominences can first be distinguished alongside the olfactory pits (TS17). The final time point (E12.5) is when the individual prominences are about to initiate fusion to form the primary palate after which they cannot be distinguished as discrete entities (TS20.3). Facial growth and morphogenesis is rapid during this period so samples were isolated at 0.5-day intervals to capture dynamic changes in gene expression.

**Figure 1:**
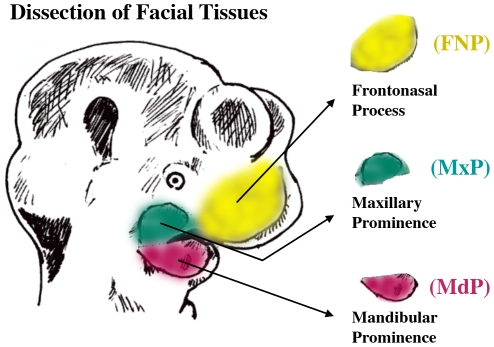
Isolation of the mouse facial prominences. Graphic representation of the embryonic mouse head illustrating the facial tissues collected for analysis.

The inbred C57BL/6J strain (Jackson Labs) was chosen for analysis to reduce genetic variation among samples and select changes in gene expression that result more from temporal- and spatial-specific differences. Moreover, the most extensive staging classification for development of the mouse face is based on this strain [10]. Following mating and visualization of a vaginal plug, pregnant females were sacrificed between E10.5 and E12.5. Mice employed for the E11, and E12 time points were housed in a reverse light cycle room to facilitate dissections at midday for all samples. Embryos were dissected from the uterus in ice-cold phosphate buffered saline solution (PBS) and then individually placed into drops of PBS in a petri dish. Embryos were staged by examination of somite numbers and craniofacial features (Table 1) and embryos conforming to the criteria for the 0.5-day interval under analysis were pooled. Pooling was necessary to obtain sufficient RNA for screening of the microarrays and also smoothes out variations between individual samples. The alternative, using fewer embryos but then incorporating a PCR amplification step, would potentially introduce considerable skewing and bias into the analysis. Table 1 indicates the number of embryos required at the various time points to obtain sufficient RNA for each prominence to be used for one microarray screening experiment. A schematic overview of the dissection process is shown in Figure 1.

**Table 1:** Staging criteria for assignment of embryos to particular timepoints and corresponding RNA yields. TS  =  Theiler stage.

Timepoint	Staging Criteria	Number of embryos needed for >5 µg total RNA from each prominence
E10.5	TS 17 *(lens vesicles form deep pockets)*	24–28
E11.0	TS 18 *(three hyoid auricular hillocks)*	8–9
E11.5	TS19 *(nares narrow to small slits)*	8–9
E12.0	TS 20.1 *(tongue begins to form)*	3–4
E12.5	TS 20.3 *(mandibular arch and the first hyoid auricular hillock begin to fuse)*	3–4

After staging, embryos were bisected with forceps at the level of the heart and the caudal portion was discarded. Tungsten needles were employed to isolate the combined maxillary and mandibular prominences and these were then separated into their individual components. Next, the medial and lateral frontonasal prominences were removed from the remainder of the head. Briefly, an incision was made in the ectoderm overlying the prominences to produce a flap of ectoderm that was peeled back with the loosely aggregated cells of the mesenchyme still attached. Note that if the cut was made too deep and extended into the forebrain region, loosely packed mesenchymal cells were no longer visible. Samples that were potentially contaminated with forebrain tissue were discarded. Suitably pure maxillary (MxP), mandibular (MdP), and frontonasal (FNP) samples were placed in separate tubes containing RNAlater (Ambion) and stored at −20°C for subsequent pooling and processing.

### RNA preparation and quality assessment

Tissue pools were dissolved in 1 ml Trizol (Invitrogen) and total RNA was extracted according to the manufacturer's instructions and resuspended in a final volume of 100 µl DEPC-treated water. Subsequently, the sample was further purified using the RNeasy Mini Kit (Qiagen, Inc.). The concentration of each total RNA sample was determined from absorbance at 260 nm (A260) and the quality of each sample was determined based on the ratio of A260 to A280. Samples with a ratio between 1.9–2.1 were considered adequately pure for cDNA synthesis. Prior to microarray analysis, individual samples were assayed using RT-PCR to ensure RNA integrity, purity, and expression of transcripts appropriate for the particular prominences. 1–2 µg total RNA was used for cDNA synthesis using random primers and Superscript III reverse transcriptase (Invitrogen) according to the manufacturer's instructions. Subsequently, 2 µl of the cDNA mixture was used for PCR in a 25 µl reaction with Accuprime Taq DNA polymerase (Invitrogen) using the following conditions: 1 cycle at 94°C for 3 min; 35 cycles at 94°C for 45 sec, 58°C for 45 sec, 68°C for 1 min; and 1 cycle at 68°C for 10 min. Specific primers (Table S1) were directed against transcripts diagnostic for the facial prominences. Representative RT-PCR results demonstrated the presence of transcripts corresponding to expression of the marker genes *Tcfap2a* (GeneID: 21418), *Bmp2* (GeneID: 12156), *Dlx2* (GeneID: 13392), and *Gsc* (GeneID: 14836) in the FNP and MxP prominences, as expected (Figure S1). We also analyzed the prominence tissues for transcripts that would indicate significant contamination from surrounding tissues, particularly the presence of adjacent CNS tissue within our FNP samples e.g. *Zic3* (GeneID: 22773) for forebrain tissue (Figure S1). Contaminated or degraded RNA samples were discarded.

### Microarray hybridization and data capture

Microarray analyses were carried out by the UCD Gene Expression Core Facility. Total RNA (2–5 µg) was converted to double-stranded cDNA (ds-cDNA) using the Superscript Choice System (Life Technologies, Inc.) and an oligo-dT primer containing a T7 RNA polymerase promoter (Genset Corp.). Subsequently, the reaction mixture was extracted with phenol:chloroform:isoamyl alcohol and the ds-cDNA was recovered by ethanol precipitation. *In vitro* transcription (IVT) was performed to generate biotin-labeled cRNA using an RNA Transcript Labeling Kit (Enzo. Inc.) and 3.3 µL ds-cDNA template in the presence of a mixture of biotin-labeled ribonucleotides. Biotin-labeled cRNA was then purified using an RNeasy affinity column (Qiagen) and the cRNA was fragmented to ensure optimal hybridization to the oligonucleotide array. Fragments between 35–200 bp in length were generated by incubating the cRNA at 94°C for 35 min in a fragmentation buffer. The quality of total RNA, labeled cRNA and fragmented cRNA were assessed using an Agilent 2100 Bioanalyzer (Agilent Technologies, Palo Alto, CA). Only samples that passed all tests of quantity and quality were used for hybridization to microarrays. The sample was then added to 200 µl of hybridization solution containing 100 mM MES, 1 M NaCl, and 20 mM EDTA in the presence of 0.01% Tween 20 to give a 0.05 µg/µL final concentration of fragmented cRNA. Next, the cRNA was hybridized to Affymetrix GeneChip® Mouse430 2.0 microarrays, which contain probes corresponding to 39,000 mouse transcripts, at 45°C for 16 hr in a GeneChip® Hybridization Oven 640 (Affymetrix, Santa Clara, CA.). The microarrays were washed and stained using an Affymetrix Fluidics Station 450 according to the manufacturer's standard protocol and scanned using the Affymetrix GeneChip Scanner 3000 (Affymetrix). Raw image data from array scans was processed in the GeneChip® Operating Software (Affymetrix) using the option in which a scaling factor was applied to bring the average intensity for all probes on the array to the same target intensity value (TGT) of 500. This normalization allowed samples to be compared across arrays. All data from our analysis are MIAME compliant, and the raw data (non-log_2_ transformed) are available via GEO (http://www.ncbi.nlm.nih.gov/geo) with the accession number GSE7759.

### *In situ* hybridization

We amplified genes chosen for verification analysis from total RNA by RT-PCR using primer pairs (Table S1) as described above. Subsequently, the products were cloned using Zero Blunt TOPO vector methodology (Invitrogen) and sequenced to confirm identity. Digoxigenin-UTP labeled probes were generated from linearized plasmids with either T7 or SP6 RNA polymerase (Roche). Whole-mount *in situ* hybridization was then performed essentially as described using staged C57BL/6J embryos [12]. Briefly, mouse embryos were fixed overnight in 4% PFA (paraformaldehyde) at 4°C, dehydrated in a graded series of methanol buffered with PBT (PBS containing 0.1% Tween 20) and stored at −70°C in 100% methanol. After rehydration, embryos were incubated overnight at 70°C in 1 ml hybridization buffer (50% formamide, 5X SSC, 50 µg/ml yeast tRNA, 1% SDS, 50 µg/ml heparin and 1 µg digoxigenin-labeled sense or anti-sense probe). After washing to remove unhybridized RNA probe, AP-conjugated (alkaline phosphatase) anti-digoxigenin antibody (Roche) was used to detect the digoxigenin-labeled RNA probe. BM purple AP substrate (Roche) was used for colorimetric detection. Images were taken using a Spot II digital camera (Diagnostic Instruments) and processed using Adobe Photoshop.

### Data filtering

To improve statistical power in light of necessary corrections for multiple testing, two filters were first applied to the dataset to discard probe sets that did not vary across any of the conditions. The first filter, the median filter [13], [14], discarded probe sets whose standard deviation across all 105 samples - using the GeneChip® Operating Software (Affymetrix) Signal values - was less than, or not significantly different than, the median standard deviation, leaving 20,868 of the original 45,101 probe sets. Selection of the median variance as a cutoff is based on the assumption that fewer than half of the genes in the genome are involved in orofacial development. The second filter removed a further 634 probe sets with a Detection call of ‘Absent’ across all 105 samples. The remaining 20,234 probe sets formed the input dataset for further statistical testing.

### Statistical differences of expression

Statistically significant differential expression between any two samples was tested using the limma package [15] of Bioconductor [16] on the log_2_-transformed Signal data. The limma package uses an empirical Bayes approach to create a moderated t-statistic by shrinking the estimated sample variances towards a pooled estimate thereby allowing more stable inference than an ordinary t-statistic when the sample size is small. P-values were calculated for each time point among different prominences (12 in total) as well as among adjacent pairs of time points for each prominence (30 in total). For all analyses, the false discovery rate was set to 1% with the additional requirement of at least a 2-fold change to be deemed significant (setting fdr = 0.01 and lfc = 1 in the decideTests function).

Differential expression across the entire time series was characterized by the trajectory clustering algorithm [14]. In lieu of ANOVA or Kruskal-Wallis p-values used by the original algorithm, the moderated t-statistic p-values from limma were used with a threshold of 0.05 (retaining the requirement for at least 2-fold change).

### Functional category analysis

Probe sets were mapped to MGI identifiers using information from the Affymetrix netaffx web interface (downloaded 18^th^ Dec 2007). Each MGI was then mapped to terms from: the Gene Ontology (GO) Biological Process and Molecular Function ontologies [17] using the association file available from the GO website (downloaded 11 May 2007); InterPro categories [18] using the MRK_InterPro.rpt file from the Jackson Laboratory website (downloaded 22 Aug 2006); the Mammalian Phenotype Ontology (MP) terms using the MGI_PhenoGenoMP.rpt file from the Jackson Laboratory website (downloaded 10 Oct 2006) and; the Kyoto Encyclopedia of Genes and Genomes (KEGG) [19] pathways using the mapping files from the KEGG website (downloaded 20 June 2007). Over-representation of each term was tested using the binomial distribution (an efficiently computable and close approximation to the hypergeometric distribution when the number of items to choose among is large [20]). P-values were adjusted using the false discovery rate (fdr) multiple comparison correction [21] and setting the threshold to 0.05.

## Results

### The Facial Transcriptome

A comprehensive, high-quality gene expression dataset was derived for mouse orofacial development by sampling the mandibular (MdP), maxillary (MxP) and frontonasal (FNP) facial prominences at 0.5-day intervals from E10.5-E12.5, after which point the prominences fused and were no longer separate entities. Seven independent biological replicates were used for each sample, which provided very strong statistical power - a false reading should only be obtained at the rate of 0.06%. In the remainder of the results, we first assess the numerical and biological quality of the dataset. We then provide an overview of gene expression profiles for genes involved in key biological processes, such as cell cycle regulation, and transcription. Lastly, we describe the identification of several genes which either: 1) had not been linked with orofacial development previously, but had been studied in the context of other biological systems; or 2) had little or no prior functional information available, but for which the dataset now suggested a role in orofacial development.

### Overview of the Expression Data and Data Quality Assessment

**Figure 2:**
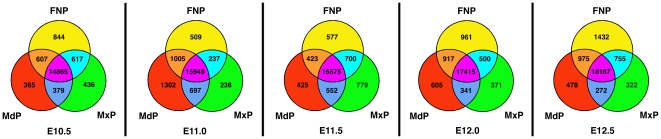
Gene expression during mouse facial development. Venn diagrams showing the number of genes expressed (called Present or Marginal) in the three facial prominences at the five developmental time points analyzed. Values represent the number of probes on the Affymetrix Mouse 430 2.0 array detected by the software in all seven replicates, i.e., called Present or Marginal using the GeneChip Operating Software.

Determination of the number of genes expressed in the dataset was based upon the Detection calls made by the Affymetrix analysis software and any “non-Absent” calls were considered in the expressed category. Using this criterion, more than 20,000 probes were expressed during the period of face formation from E10.5–E12.5. Of 45,101 total probes on the Affymetrix Mouse 430 2.0 microarray, 30% had detectable expression in all 105 samples while 47% were undetectable in any sample. In terms of spatial distribution, 80–82% of the expressed sequences were detected in all three regions of the developing face (Figure 2). A more limited number of probes were expressed in only one prominence or were shared between two of the three prominences (Figure 2). In addition to expression defined by Detection calls, a stringent assessment of prominence-specific expression was obtained by applying the limma package [15] of BioConductor [16] to the Signal values at each time point. Of 20,234 probes tested, 1,506 displayed at least one statistically significant difference between prominences for a given time point. The minimum observed was 171 differences between the MdP and MxP at E10.5, with a maximum of 646 differences in sequence intensity between MdP and FNP at E12.5 (Figure S2A). In general, an increasing number of differences occurred between individual prominences at each successive time point consistent with a divergence of function accompanied by greater complexity of gene expression (Figure S2A). The greatest number of statistically significant prominence-specific gene expression differences occurred between the MdP and FNP, and the least between the MdP and MxP, at all time points. With respect to temporal changes in gene expression, 1,328 probe sets displayed at least one significant increase or decrease between adjacent time points within a particular prominence. This ranged from a minimum of 44 significant changes for the FNP between E11.5 and E12.0, to 582 differences for the MxP between E11.0 and E11.5 (Figure S2B). The data potentially illustrate both an earlier onset of significant gene expression changes in the MdP and circadian aspects to the changes that are occurring in the three prominences. A more in-depth characterization of genes differentially expressed over the entire time series is given later in this section.

Reproducibility of the qualitative Detection calls, namely Absent, Marginal and Present, were assessed using the kappa statistic which measures agreement among categorical variables [22] with excellent reproducibility denoted by kappa values >0.75. The median kappa value among replicates was 0.7938 (min = 0.65, max = 0.84). Relatively high kappa values also occurred among non-replicates (median = 0.7649, min = 0.58, max = 0.83) indicative of considerable similarity in global gene expression across the three prominences at all time points. This latter finding suggested that there were unlikely to be widespread radical differences in gene expression between the prominences over the time course of the analyses. Instead, the data was more consistent with subtle alterations in the expression of multiple genes potentially coupled with extensive differences in a limited set of transcripts.

**Figure 3:**
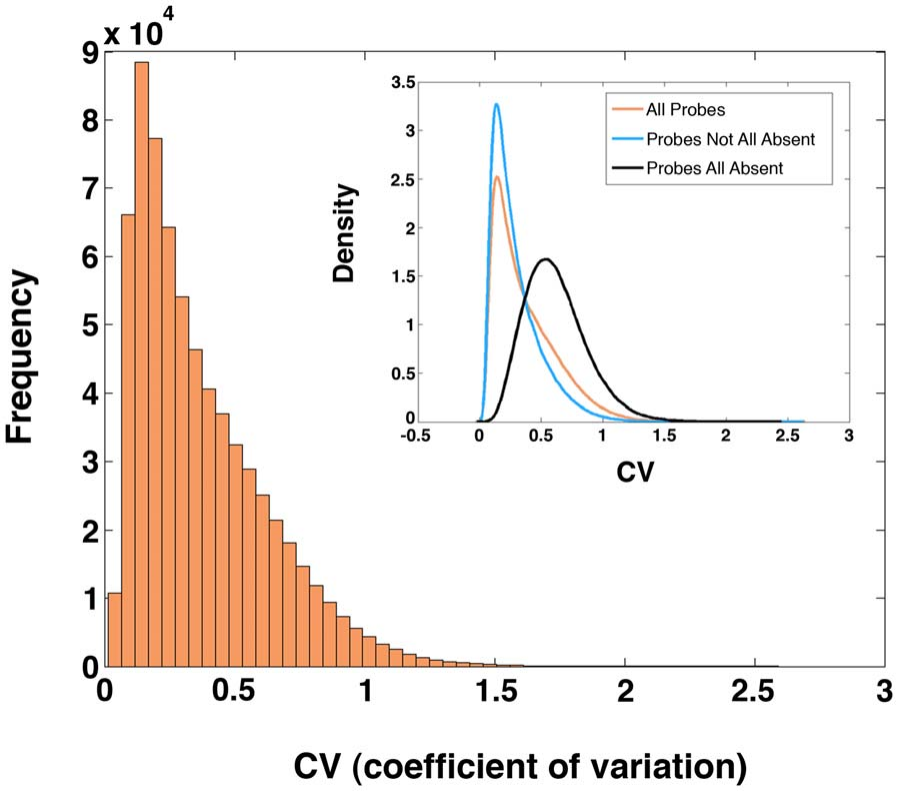
Coefficient of variation (CV) analysis demonstrates high quality of the dataset. The main graph illustrates the CV distribution for all probes tested. In the inset graph, the data are represented by the brown line. Removal of the “probes all absent” calls (black line), which show greater signal variation, indicates that the “Present” and “Marginal” probes have even greater reproducibility, illustrated by the shift in the CV curve.

Reproducibility of the quantitative Signal values was assessed using the coefficient of variation (CV), defined as the ratio of the standard deviation to the mean [22]. The CV is zero for perfect reproducibility while higher values indicate poorer reproducibility. If the CV is close to 1, the variability of measurement is on the same scale as the signal and no meaningful analysis is possible [23]. In our dataset the median CV was 0.30 among replicates (min = 0.01, max = 2.59), calculated over all probes in all prominence- and time-specific replicate groups (Figure 3). Much of the high variability was attributable to probes called Absent in all 105 samples (Figure 3, inset), an important distinction since data for all-Absent probes were removed in any further analyses. Disregarding those probes called Absent in all samples, the median CV dropped from 0.30 to 0.24, versus CV = 0.59 among all-Absent probes. The dramatic effect seen when distinguishing among probes based on Absent calls highlights not only the benefits of incorporating the Detection information, but also the importance of utilizing the kappa statistic to assess the quality of that Detection information. High quality of replication in terms of the mean and standard deviation among replicates for each sample was also observed (Figure S3).

### Biological Verification of the Dataset

**Figure 4:**
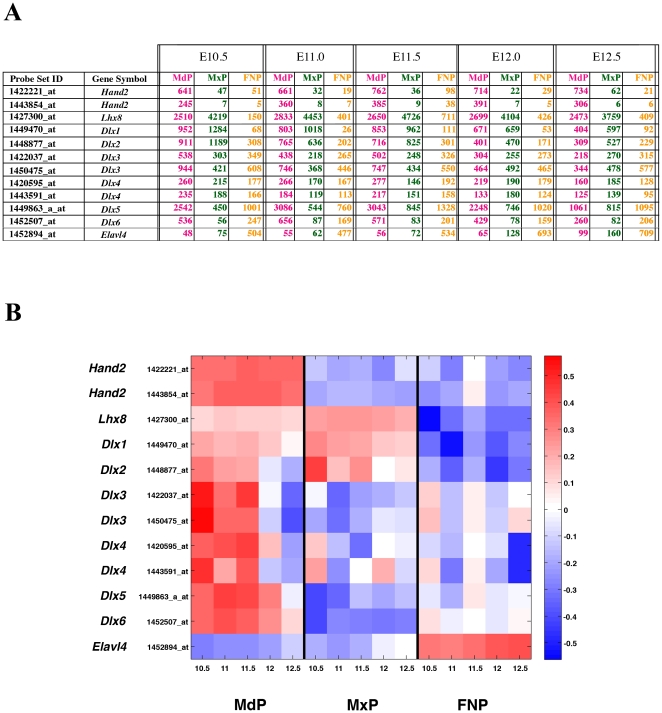
Biological verification of the dataset. (**A**). Raw data for the prominence-specific genes and associated probe sets (left) indicated at the five time points in the three prominences. (**B**). Corresponding heatmap for the prominence-specific genes and associated probe sets. The heatmaps show the expression data for each probe scaled such that the vector of log_2_ expression values for a probe (averaged among replicate samples per time point) has a mean of zero and a magnitude of one. Red and blue indicate high and low expression, respectively.

For initial biological verification we focused on genes with established prominence-specific expression patterns such as *Hand2* (GeneID: 15111), *Lhx8* (GeneID: 16875), *Elavl4* (GeneID: 15572) and members of the *Dlx* family [24], [25], [26], [27]. There was excellent agreement between our microarray data (Figure 4A and B) and the expression patterns of these genes as previously determined by whole mount *in situ* hybridization analysis (WMISH). Thus, the microarray data demonstrated that *Dlx1* (GeneID: 13390), *Dlx2* and *Lhx8* (also known as *Lhx7*) were preferentially expressed in the MdP and MxP (Figure 4A and B) in agreement with published WMISH results [25], [26]. Previous data had also documented that *Dlx3* (GeneID: 13393), *Dlx4* (GeneID: 13394), *Dlx5* (GeneID: 13395) and *Dlx6* (GeneID: 13396) were expressed most highly in the MdP, and this was again reflected at the earlier time points in our microarray data (Figure 4A and B). Of note, signal levels were also detectible for these latter four *Dlx* genes in the FNP and MxP, a finding that was consistent with *in situ* hybridization data ([26] and data not shown). A similar concordance was found between our data and the WMISH expression patterns for *Hand2* (also known as *dHand*) a gene that was expressed strongly in the MdP, but not in the MxP and FNP (Figure 4A and B). We chose *Elavl4* (*Hud*) as a marker that should be enriched in the FNP as this gene is associated with neurogenesis and is expressed in the developing olfactory epithelium of the nasal pit [27]. The microarray data again reflected appropriate FNP-specific expression for *Elavl4* (Figure 4A and B). *Elavl4* is also strongly expressed in the developing peripheral nervous system, including the trigeminal ganglia, but we did not detect significant levels of *Elavl4* expression in our MdP and MxP samples until E12. This finding provided additional evidence that our prominence samples were not heavily contaminated with surrounding craniofacial tissue. Thus, our dissections produced prominence specific material, and there was an excellent correspondence between our microarray findings and established differential gene expression patterns.

### Functional Category Analysis of the Temporal Expression Differences

Analysis of over-represented terms among probes with temporal expression differences supported a model in which processes such as cell metabolism and cell growth were declining while differentiation was increasing. Alterations in expression between adjacent time points across the entire time series were characterized using the trajectory clustering algorithm [14]. This approach generates a set of direction change labels between adjacent time points, namely increased, flat, or decreased expression, that can then be extended into a more informative matrix by including all time points. Data collection at five time points generated a total of 81 possible trajectories for each prominence (Table S2). The majority of probes, 15,510 out of 20,234 tested, exhibited consistently flat profiles (no changes) across all five time points in the three prominences. The remaining set of 4,724 probes showing differential expression over time in at least one prominence was much larger than the set of 1,323 probes found using limma alone on adjacent time points, since trajectory clustering captures changes that unfold over longer periods of time.

**Figure 5:**
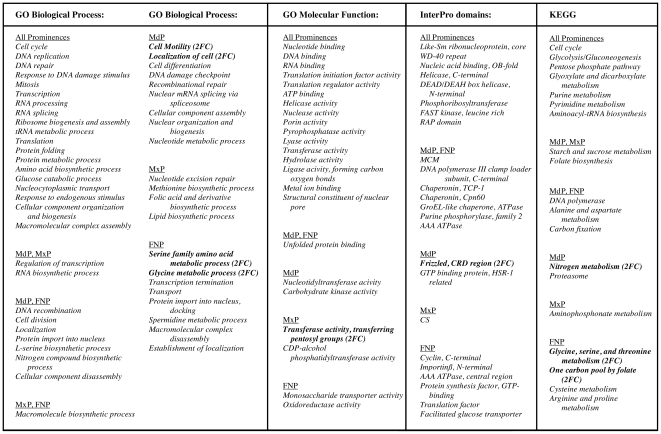
Functional analysis of trajectories: selected categories showing at least two decreases and no increases over the time course. Over-represented terms, reaching significance at fdr  = 0.5 for the binomial distribution, were based on probes achieving limma statistical significance. Those shown in bold and labeled “(2FC)” reach the additional criterion of a 2-fold change in gene expression.

Our initial analysis concentrated on trajectories with predominantly upward or downward trends. Examination of the trajectories showing at least two decreases and no increases over the time course revealed a broad and statistically significant down-regulation of genes involved in cell division and general metabolism in all three prominences between E10.5–E12.5 (Figure 5 and Tables S3A-J). Seven functional categories associated with cell motility, amino acid metabolism, and other metabolic processes showed a consistent 2-fold reduction in expression during the time course while genes involved with cell cycle, splicing, and translation showed analogous declines in the expression, although less than 2-fold (Figure 5).

Across the prominences, ∼28% of the down-regulated genes were involved in protein metabolic processes, 16% in biosynthetic processes, 18% in transcription, 10% in RNA processing, 8% in translation, 11% in the cell cycle, and 3% in nucleocytoplasmic transport. With respect to individual categories, 25–35% of the genes assigned to glucose metabolism, nucleotide metabolism, DNA replication, DNA repair, RNA processing, amino acid activation, and nucleocytoplasmic transport were found to decrease in expression in the three prominences. Certain terms, including DNA replication initiation, did show a wider range of the percentage of affected genes (Table S3E). The variation in the number of down-regulated genes within such categories may indicate subtle differences in developmental programs among the prominences. Similarly, the assignment of particular terms to individual prominences - or to two of the three prominences - highlights further pathways that may contribute to differential development (Figure 5). In general, though, the relatively small magnitude of the observed gene expression decreases, and the broad range of metabolic functions involved likely reflect an equivalent slowing of growth across all three prominences rather than a drastic change in basic cellular processes.

**Figure 6:**
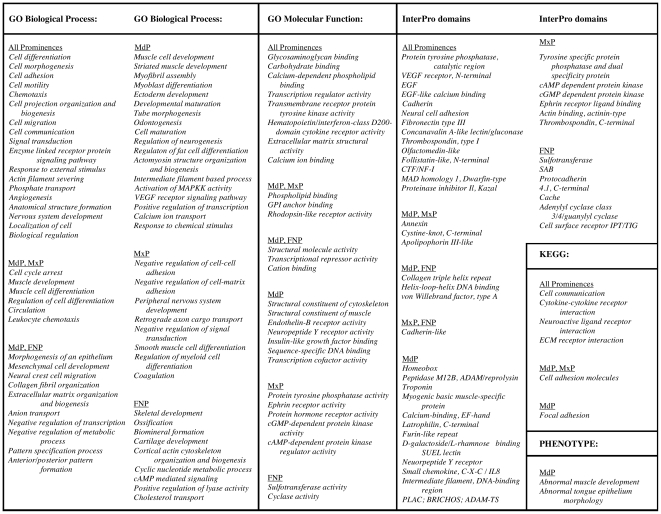
Functional analysis of trajectories: selected categories showing at least two increases and no decreases over the time course. Over-represented terms, reaching significance at fdr  = 0.5 for the binomial distribution, were based on probes achieving limma statistical significance. All reach the additional criterion of a 2-fold change in gene expression.

Conversely, for genes showing an upward trend in expression for at least two of the four intervals and no decreases, the over-represented terms reflected differentiation (Figure 6 and Tables S3K-U). These increases were more robust, and consistently exceeded the criterion of a 2–fold change in the level of expression. Prominent categories displaying expression increases in all three prominences included cell adhesion, cell migration, and cell morphogenesis. Categories for transcription factors, cell signaling, protein interaction domains, calcium-dependent processes, and the extracellular matrix were also highlighted in the various prominences. Notably, ∼50% of the genes associated with phosphate transport showed increasing expression in all prominences (Table S3Q), many by more than 2-fold (Table S3K).

Genes showing an upward trend in expression reflected tissue specialization into teeth, cartilage, and nervous system components with prominence-specific categories more evident than in the gene sets showing decreased expression (Figure 6 compared with 5). In particular, the MdP exhibited increased expression of genes associated with various muscle differentiation categories, reflecting tongue development. The MdP was also over-represented for terms associated with tube morphogenesis and epithelial cell differentiation, presumably reflecting development of the taste buds, teeth, and salivary glands (Figure 6). In contrast, genes associated with the peripheral nervous system and with cyclic nucleotide dependent protein kinase signaling were apparent in the MxP. The FNP was enriched for genes associated with sulfotransferase activity and skeletal development. The MdP, MxP, and FNP were also distinguished by the different protein motifs and protein:protein interaction domains that the trajectory clustering analysis highlighted in the three prominences (Figure 6). We present a further analysis of prominence specific differences in the next section.

In addition to the generally upward or downward gene expression profiles, the trajectory clustering analysis also highlighted several other terms that were over-represented in specific trajectories within a given prominence (Table S2). Two broad themes can be identified from these additional profiles. First, categories in GO Molecular Function, and Biological Process ontologies associated with oxidative phosphorylation showed fluctuating trajectories (up and down) in all three prominences. This finding suggests that energy generation during this period of embryogenesis displays periodicity, consistent with a circadian rhythm that may reflect the nocturnal activity of the mother. Second, ontological terms containing the words “histones” or “chromatin” also had fluctuating trajectories, potentially indicating dynamic changes in transcription and DNA packaging.

### Functional Category Analysis of Prominence-Specific Expression

While trajectory clustering analysis highlights dynamic changes in gene expression, this method is not well suited to visualizing instances in which expression may be higher in one of the prominences, but is not increasing or decreasing over the time course. Therefore, to obtain a prominence-specific view of expression changes we studied the set of 1,506 probe sets showing at least one significant (2-fold) difference among prominences at a given time point. This set was further divided into MdP (705 probes), MxP (495 probes), and FNP (760 probes) groups based upon differences in expression from at least one other prominence for the same time point.

**Figure 7:**
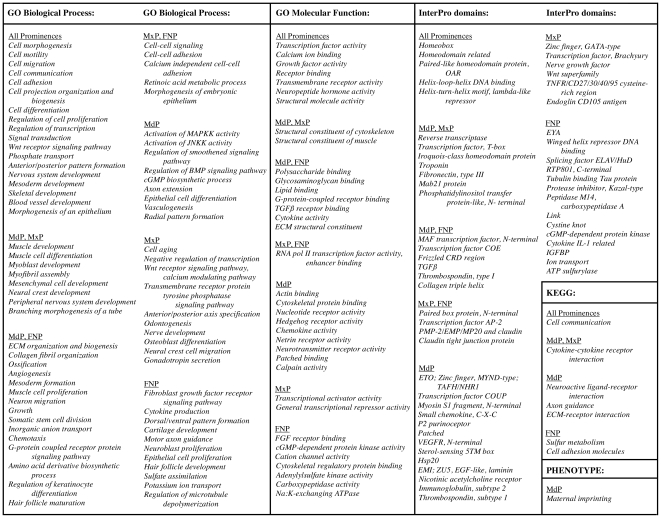
Selected categories showing increased expression in the facial prominences. Prominence-specific groups were created among probes achieving limma statistical significance, with the additional criterion of a 2-fold change in gene expression, by assigning a probe to a group if expression was increased in the group over at least one other prominence at a specific time point.

These probe set groups were then analyzed with respect to GO, InterPro, MGI Phenotype, and KEGG terms to determine over-represented categories (Figure 7 and Tables S4A-E).

Consistent with the data presented in Figure 2, in which we observed significant overlap of detectable expression among the three prominences, the functional characterization again revealed that the prominences had very similar gene expression dynamics since many of the categories observed were seen in the MdP, MxP and FNP. The data also supported our conclusions from the trajectory clustering analysis that significant changes were occurring in growth and differentiation. Alterations in RNA expression levels of transcription factors, signaling molecules, cell adhesion components, and structural proteins were apparent (Figure 7). Cell fate determination, morphogenesis, motility, migration, and adhesion were also prominent categories with ∼30% of the up-regulated genes assigned to cell differentiation, 30% to cell communication, and 10% to cell motility in each of the prominences. There were also notable prominence-specific differences in the distribution of expressed genes within particular categories that may help distinguish the ultimate fate of these separate facial regions. In this regard, 36% of all genes increasing by >2-fold within the MxP were associated with transcriptional regulation, whereas this figure was 29% and 25% for the MdP and FNP, respectively. Similarly, 16% of genes in the FNP were categorized as cell adhesion, 13% in the MxP, and only 10% in the MdP.

Progressive specialization of tissues in the prominences was highlighted by the distribution of over-represented categories associated with development of muscle, bone, cartilage, dentition, vasculature, glands, epithelia, and the nervous system between the MdP, MxP, and FNP (Figure 7). Among probes in the MdP-specific group, the over-represented terms were associated with signal transduction via MAPK and JNK second messenger pathways, as well as with Hedgehog, VEGF, BMP, chemokine, netrin, and neurotransmitter signaling. The MxP group was enriched for terms associated with development of teeth, nerves, and the endocrine system, correlating with formation of the dentition, the trigeminal ganglion, and part of the pituitary system from Rathke's pouch in the oral epithelium. Also prominent in the MxP were genes involved in Wnt signaling and transcriptional control, particularly the negative regulation of transcription. The FNP group was over-represented for categories associated with fibroblast growth factor receptor signaling, sulfate assimilation, ion transport, cell adhesion, cartilage formation, and nerve development, some of which likely reflect development of the specialized olfactory epithelium.

Over-represented categories shared between two of the three prominence groups included muscle, neural crest cell, and peripheral nervous system development in the MdP and MxP. With respect to muscle development, 6.5% and 9.5% of genes were assigned to this category in the MdP and MxP, respectively, but only 2.5% in the FNP group. TGFβ signaling, chemotaxis, angiogenesis, the extracellular matrix, and skeletal formation were highlighted in the MdP and FNP groups. In these two prominences, 3% of genes were in the ECM organization and biogenesis category, whereas the equivalent MxP dataset contained less than 1% of such genes. The MxP and FNP groups shared the categories of transcription factors, retinoic acid, cell adhesion, and claudins. Overall, these findings reflect the widespread importance of signal transduction, transcriptional regulation, and cell:extracellular matrix contacts in growth and morphogenesis of the developing face. With respect to pattern formation, it was noticeable that gene categories associated with different axes of polarity were highlighted in the three prominences. The apparent segregation of radial (MdP), anterior/posterior (MxP), and dorsal/ventral (FNP) patterning in these prominences could represent one important developmental mechanism whereby their individual identities are determined. A further point of interest is that maternally imprinted genes were clearly over-represented in the MdP compared with the MxP and FNP (Figure 7 and Table S4E). Finally, as expected, many genes we identified as expressed in our dataset were linked with the MGI Phenotype categories “cleft palate”, “midline facial cleft” “abnormal cartilage morphology” and “abnormal myogenesis” (Table S4E).

### Detailed Examination of Facial Prominence Expression by Functional Category

**Figure 8:**
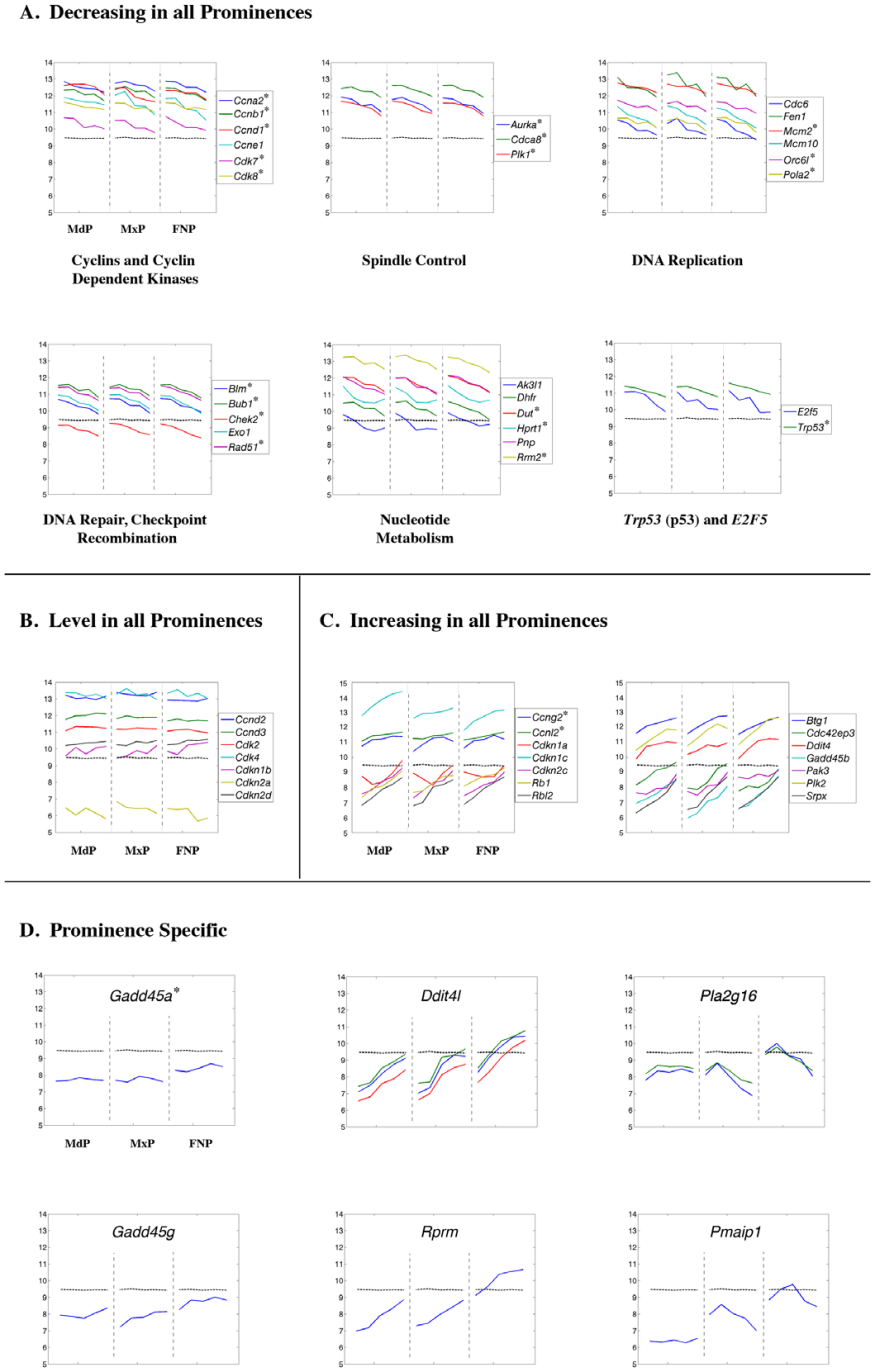
Expression profiles of cell cycle regulatory genes. Each panel shows the expression profile for the gene or genes indicated. The x-axis represents the five time points for each prominence (MdP, MxP, and FNP). The y-axis shows the relative expression level on a log_2_ scale – i.e. every integer represents a doubling in the level of expression from the preceding number. The dashed black line indicates the average expression level for all probes. The lines of other colors illustrate the data obtained for the indicated gene shown in the key at the right. If only one gene is shown in a panel the various colors indicate the data obtained from different probes corresponding to that gene. Genes marked by an asterisk in the increasing, decreasing, or prominence-specific categories have statistically significant differences in expression over the time course or between prominences but fail to satisfy the additional criterion of a 2-fold change in gene expression.

The assignment of prominence expression changes to particular GO, InterPro, KEGG or MGI Phenotype categories identified some tissue-specific differences, but these were relatively limited compared with the commonalities in cell metabolism, cell growth, and differentiation. Therefore it was necessary to study gene expression changes within a particular category, such as transcription and cell adhesion, in greater detail to identify critical prominence-specific differences. Accordingly, we examined individual genes involved in cell cycle progression, general metabolism, the cytoskeleton and cell adhesion, the extracellular matrix, transcription, and chromatin dynamics. In each category, genes were divided into those that were coordinately regulated in all three prominences (level, increasing, or decreasing) and those that were differentially regulated. With the exception of genes in the “level” group that were coordinately regulated, all the examples presented were identified as differentially expressed by our statistical analyses. Moreover, we concentrated on genes that displayed at least a 2-fold expression difference between the first and last time point. The exceptions, genes and probe sets that are classified as increasing, decreasing, or prominence-specific, but which failed to fulfill this additional 2-fold expression difference criterion, are marked in Figures 8–19 with an asterisk.

**Figure 9 pone-0008066-g009:**
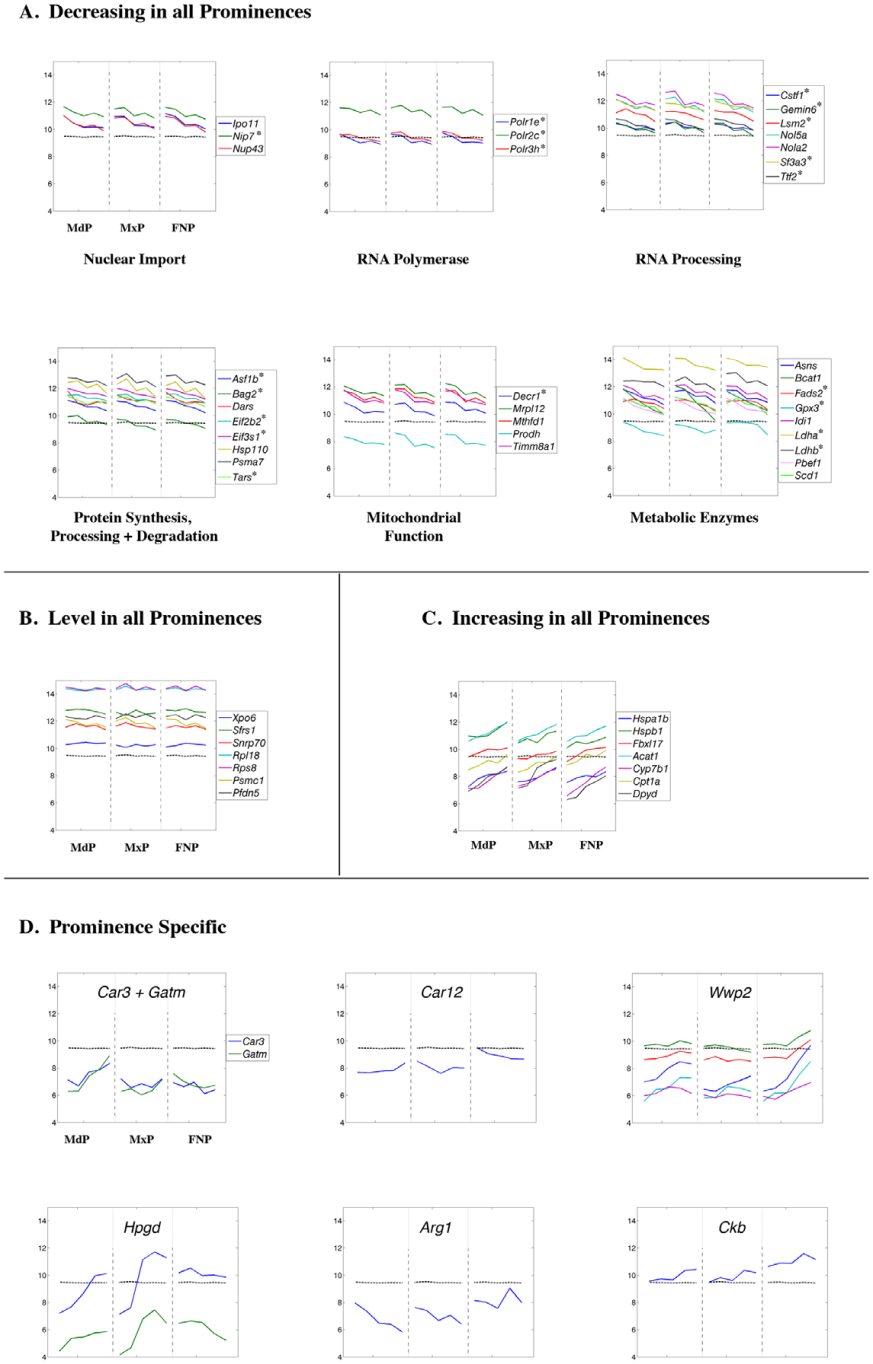
Expression profiles of genes involved in general cellular metabolism. Legend as in Figure 8.

**Figure 10 pone-0008066-g010:**
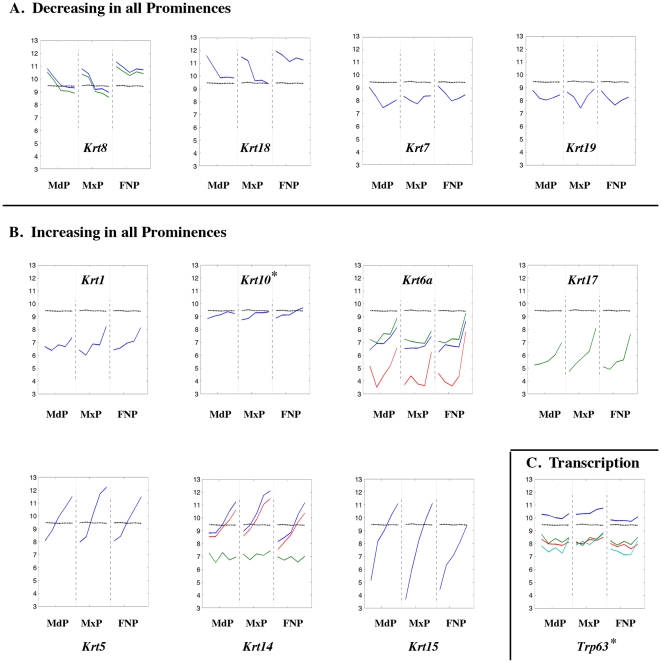
Expression profiles of keratin genes. Legend as in Figure 8.

**Figure 11 pone-0008066-g011:**
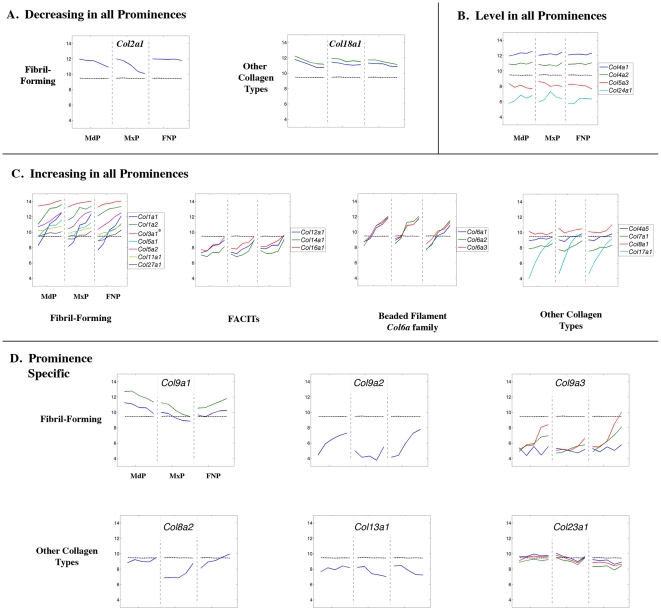
Expression profiles of collagen genes. Legend as in Figure 8.

**Figure 12 pone-0008066-g012:**
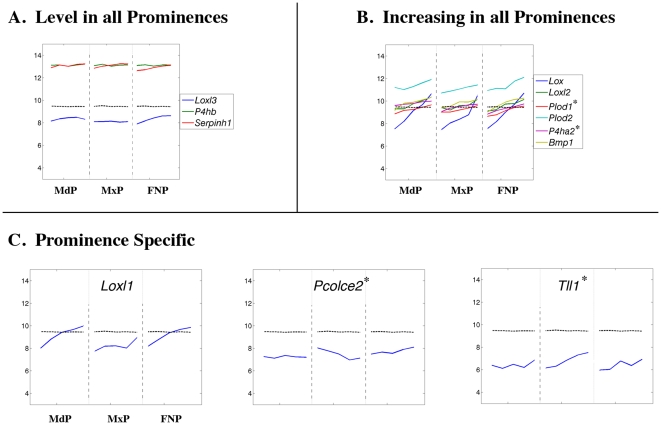
Expression profiles of genes encoding collagen processing enzymes and chaperones. Legend as in Figure 8.

**Figure 13 pone-0008066-g013:**
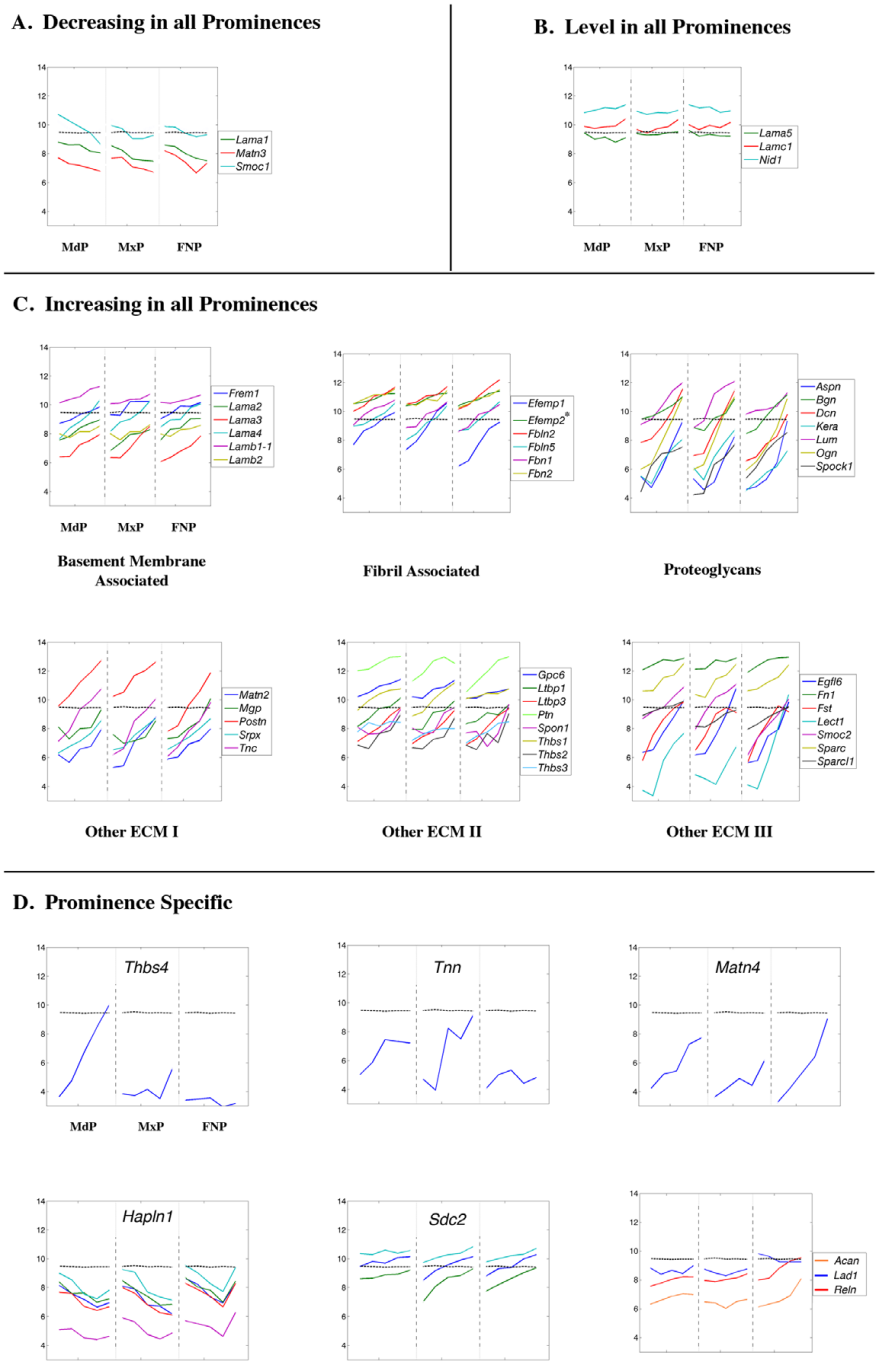
Expression profiles of structural genes of the extracellular matrix. Legend as in Figure 8.

**Figure 14 pone-0008066-g014:**
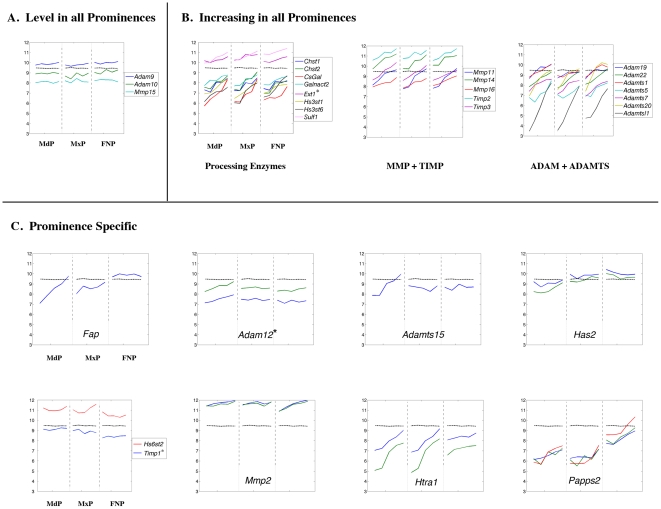
Expression profiles of genes encoding extracellular matrix modifying components. Legend as in Figure 8.

**Figure 15 pone-0008066-g015:**
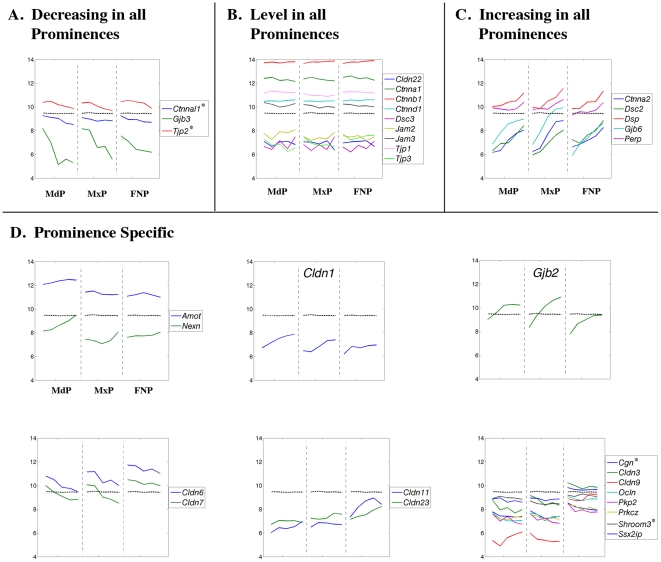
Expression profiles of genes encoding junctional complex proteins. Legend as in Figure 8.

**Figure 16 pone-0008066-g016:**
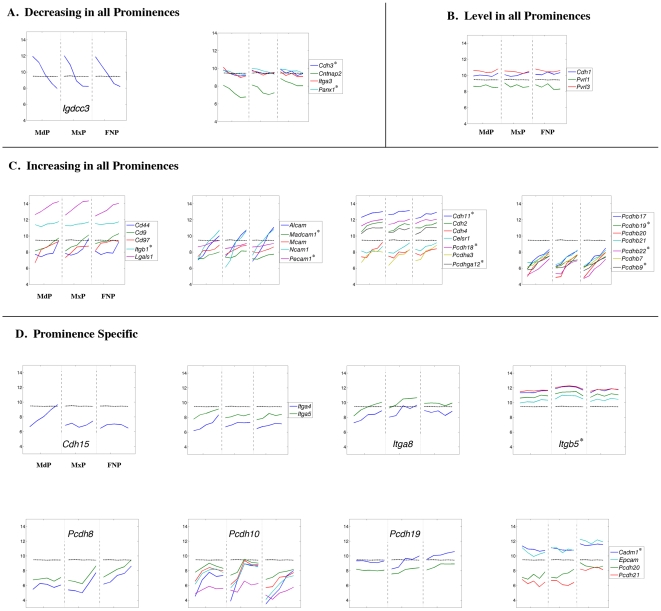
Expression profiles of genes encoding cell adhesion molecules. Legend as in Figure 8.

**Figure 17 pone-0008066-g017:**
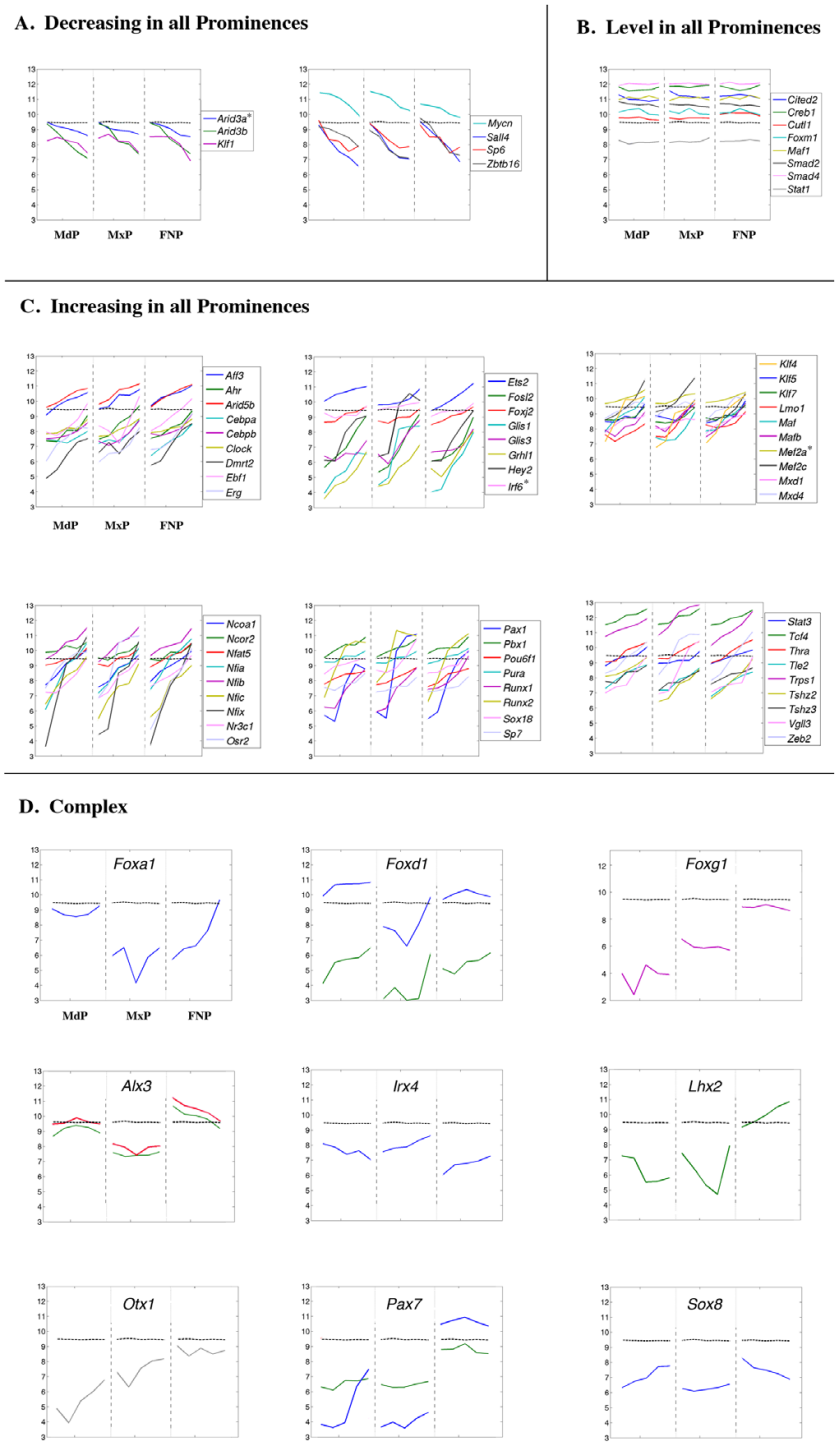
Expression profiles of genes encoding transcription factors with coordinately regulated (A–C) or complex (D) patterns. Legend as in Figure 8.

**Figure 18 pone-0008066-g018:**
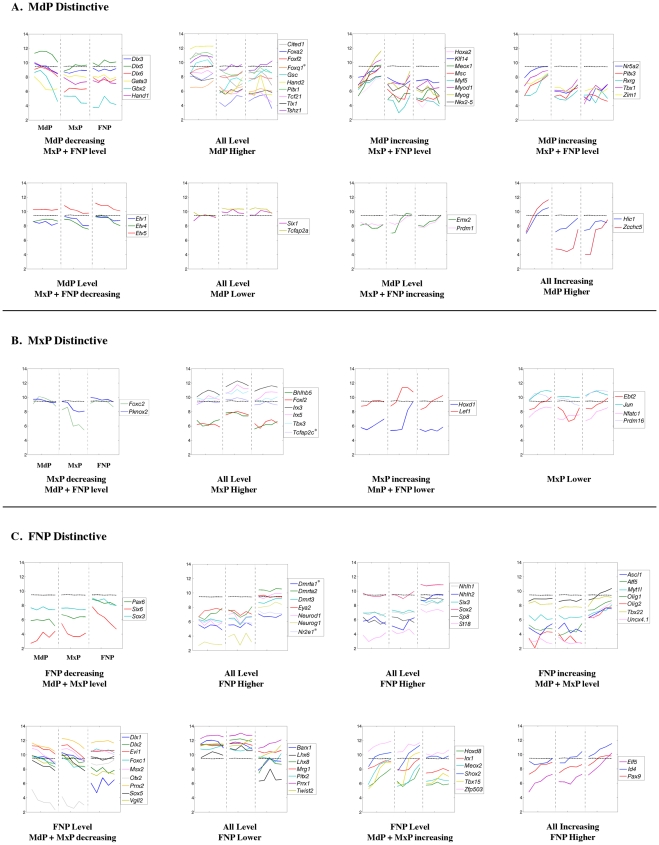
Expression profiles of genes encoding transcription factors with coordinate regulation in two of the three prominences. Legend as in Figure 8.

**Figure 19:**
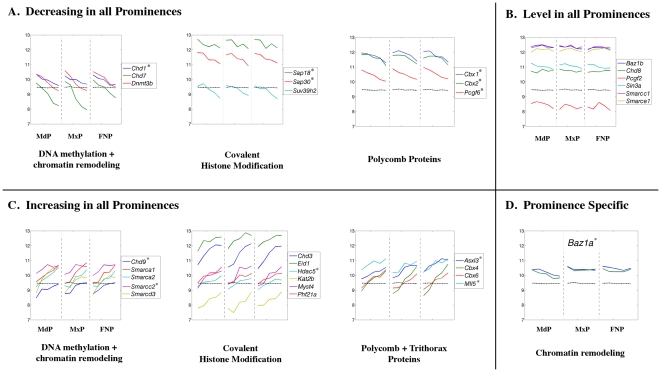
Expression profiles of genes encoding chromatin binding and remodeling factors. Legend as in Figure 8.

### Genes involved in Cell Cycle Progression and General Metabolism

Analysis of genes involved in replication and cell division is shown in Figure 8A–D. In agreement with the Gene Ontology analysis there was an ∼2-fold reduction in the transcript levels of multiple genes involved in promoting cell cycle progression in all prominences (Figure 8A). Such genes included many cyclins and cyclin dependent kinases as well as genes required for nucleotide metabolism, DNA replication, DNA repair, and cell division. Concurrently, transcript levels for genes involved in the negative regulation of cell cycle progression showed a tendency to increase (Figure 8C). Transcripts encoding Rb1 (GeneID: 19645), the G2 checkpoint kinase Plk2 (GeneID: 20620), and several cyclin dependent kinase inhibitors (cdki/cdkn), rose 2 to 4-fold in the prominences. A general increase in expression was also noted for the DNA damage response gene *Gadd45b* (GeneID: 17873), for *cdc42ep3* (GeneID: 260409), a negative regulator of *cdc42*, (GeneID: 12540) and for *Cdkn1c* (GeneID: 12577), which encodes p57 cdki. There were some modest prominence-specific differences in *Cdkn1c* expression, though, with the FNP displaying ∼2-fold lower transcript levels at E10.5 and the MdP having 3 to 4-fold higher levels by E12.5. Nevertheless, the overall similarity of the expression profiles for multiple cell cycle associated genes in the three prominences is striking and suggests coordinate regulation. We note that there was a small subset of such genes that displayed prominence-specific gene expression (Figure 8D). In the FNP, transcripts for *DDit4l* (GeneID: 73284), *Pla2g16* (GeneID: 225845), *Rprm* (GeneID: 67874), *Gadd45a* (GeneID: 13197), and *Gadd45g* (GeneID: 23882) were found at 2 to 4-fold higher levels than in the other prominences. Moreover, expression of *Pmaip1* (GeneID: 58801), a gene involved in the induction of apoptosis that is also termed *Noxa* [28], was 4 to 8-fold lower in the MdP and remained relatively constant throughout the analysis, whereas in the MxP and FNP the *Pmaip1* expression profiles were more dynamic, peaking and then decreasing.

Concurrent with decreased expression of genes involved with replication, we also observed ∼2-fold reductions in the expression of multiple genes associated with basic cellular processes, including nuclear import/export, transcription, RNA processing, and RNA degradation, in all three prominences between E10.5 and E12.5 (Figure 9A). Similar transcript reductions were observed for several genes required for ribosome biogenesis, protein synthesis, protein folding, protein degradation, and aspects of mitochondrial function (Figure 9A). Transcripts for many other genes involved in these basic processes remain essentially level during this developmental period (Figure 9B). Only a few genes displayed a slight increase in transcript levels, including several involved in ubiquitination, as well as the catabolism of nucleotides, fatty acids and cholesterol (Figure 9C). There was also an MdP-specific increase in transcript levels for enzymes associated with skeletal muscle function, including *Car3* (GeneID: 12350) and *Gatm* (GeneID: 67092) (Figure 9D), reflecting the development of the tongue and masticatory muscles from this prominence [29]. Taken together, though, these observations indicate that there is a simultaneous overall reduction in the rate of growth and in many aspects of metabolism in all three facial prominences from E10.5–E12.5.

### Keratin Genes

We next examined whether expression of genes involved in differentiation were activated to counter-balance the decreased expression of those associated with replication. Several classes of genes were chosen to study the process of differentiation, including intermediate filament proteins (Figure 10), extracellular matrix molecules (Figures 11–14), cell adhesion molecules (Figures 15, 16), and transcription factors (Figures 17, 18). As described in detail below, these studies demonstrated that the three sets of prominences exhibited striking commonalities in the onset and progression of differentiation. We focused initially on keratin genes (Figure 10A, B) because their expression provides an excellent readout of development of the surface ectoderm from a simple to multilayered epithelium [30]. Over the time course, there was a 3 to 4-fold reduction in *Krt8* (GeneID: 16691) and *Krt18* (GeneID: 16668) transcripts, which are associated with simple embryonic epithelia, and the levels of *Krt7* (GeneID: 110310) and *Krt19* (GeneID: 16669) transcripts also dropped initially before recovering (Figure 10A). Concurrently, there was a rise in the transcript levels for keratins associated with more mature stratified epithelia (Figure 10B). Transcripts derived from *Krt5* (GeneID: 110308), *Krt14* (GeneID: 16664), and *Krt15* (GeneID: 16665), which are expressed in the basal epithelial layers, rose exponentially from E10.5 onwards. *Krt1* (GeneID: 16678) and *Krt10* (GeneID: 16661) transcripts, associated with the suprabasal epidermal layers, were increasing more slowly during the same period. Lastly, *Krt6a* (GeneID: 16687) and *Krt17* (GeneID: 16667), which are expressed in the suprabasal layers of epithelia of the oral cavity, including the tongue [30], began to rise at the later time points.

The switch from keratin genes expressed in simple epithelia to those present in stratified epithelia occurred simultaneously in the three prominences. Nevertheless, evident differences in the three prominences were superimposed on this overall pattern (Figure 10A, B). In the FNP the switch between keratins associated with simple (*Krt8* and *Krt18*) versus complex (*Krt15*) epithelia progressed at a much slower rate. The MxP also displayed differences from the other prominences, especially in the levels of *Krt5* and *Krt14* expression, which were higher, and the expression profiles of *Krt6a* and *Krt7*, which were less dynamic. MdP specific differences were less pronounced. Overall, though, each prominence exhibited a unique molecular fingerprint of keratin gene expression. These prominence-specific differences were also reflected in the expression of *Trp63* (GeneID: 22061), encoding the p63 transcription factor that promotes the commitment to epithelial stratification [31]. Transcripts encoding p63 were lowest in the FNP reflecting the slower rate of change between keratins characteristic of simple versus stratified epithelia in this prominence (Figure 10C).

### Genes Encoding Components of the Extracellular Matrix

#### Collagens and the Collagen Processing Machinery

Transcripts encoding many components of a differentiated extracellular matrix were rapidly increasing during the same period in all prominences, typified by many collagen gene family members [32], [33]. Though expression of a small number of collagen genes decreased over time, or remained level (Figure 11A, B), a majority of these genes displayed co-ordinate up-regulation of transcript levels in the three prominences (Figure 11C and Figure S4). Several of these genes, including *Col1a2* (GeneID: 12843), *Col3a1* (GeneID: 12825), and *Col5a2* (GeneID: 12832), encoding fibril-forming collagens, already had high transcript levels in all prominences by E10.5, and their expression continued to increase by up to 6-fold by E12.5. Another set of collagen genes had lower expression at the outset of the time course, but then transcript levels rose coordinately through E12.5 in the MdP, MxP, and FNP. These genes include those encoding fibril-forming collagens (*Col1a1*, GeneID: 12842; *Col5a1*, GeneID: 12831; *Col27a1*, GeneID: 373864), fibril associated collagen (*Col16a1*, GeneID: 107581), and structural “beaded filament” collagens (*Col6a 1*, GeneID: 12833; *Col6a2*, GeneID: 12834; *Col6a3*, GeneID: 12835). This increase was most dramatic for the membrane associated collagen *Col17a1* (GeneID: 12821), for which levels rose >30-fold between E10.5–E12.5. The rapid increase in transcript levels for this type XVII collagen is presumably indicative of desmosome formation associated with the development of the stratified epithelia of the skin and oral cavity [32], [33].

A subset of collagen genes, particularly *Col4a1* (GeneID: 12826) and *Col4a2* (GeneID: 12827), encoding fibril-forming collagens, were expressed at high but generally flat levels in all prominences throughout this period of orofacial development (Figure 11B). Only two collagens, *Col2a1* (GeneID: 12824) and *Col18a1* (GeneID: 12822), showed a downward trend of expression in all prominences (Figure 11A). *Col2a1* transcript levels started high and then dropped up to 4-fold during this developmental period, although this reduction was less pronounced in the FNP. This observation reflects down-regulation of *Col2a1* during the transition from chondrocyte precursors to differentiated cells. Collagen type XVIII is a component of some basement membranes, but also generates the peptide endostatin that inhibits endothelial proliferation [32], [33]. A reduction in *Col18a1* expression would affect both functions, and potentially stimulate angiogenesis in the prominences.

Although there was considerable evident coordinate regulation of collagen gene expression, there were also clear prominence-specific differences (Figure 11D). The FNP was distinguished by increasing *Col9a1* (GeneID: 12839) transcript levels, compared with decreasing levels in the MdP and MxP. The FNP also had the highest *Col9a3* (GeneID: 12841) transcripts levels by E12.5, with intermediate levels seen in the MdP and low levels in the MxP. Distinctive expression profiles in each prominence were also observed for *Col8a2* (GeneID: 329941), and *Col23a1* (GeneID: 237759) (Figure 11D). In the MxP, *Col9a2* (GeneID: 12840) transcript levels remained relatively flat throughout the time course, whilst this gene showed increasing levels in the other prominences (Figure 11D). For the MdP, there was a slight increase in *Col13a1* (GeneID: 12817) levels, whereas they decreased ∼3-fold in the MxP and FNP (Figure 11D). The significant prominence-specific differences in expression apparent for this group of collagen genes, coupled with more subtle expression differences for *Col4a5* (GeneID: 12830), *Col6a1*, *Col8a1* (GeneID: 12837), *Col11a1* (GeneID: 12814), *Col12a1* (GeneID: 12816), and *Col14a1* (GeneID: 12818) (Figure 11C), will likely generate a specific extracellular matrix in each facial prominence.

The production of mature collagen proteins also requires several processing enzymes and specific chaperones [32], [33]. We observed high but relatively constant expression levels for several genes necessary for collagen modification and assembly (Figure 12A; *P4hb*, GeneID: 18453; *Serpinh1*, GeneID: 12406), while expression of others increased 2 to 8-fold (Figure 12B; *Bmp1*, GeneID: 12153; *Lox*, GeneID: 16948; *Loxl2*, GeneID: 94392; *Plod2*, GeneID: 26432) in concert with collagen gene expression. Limited prominence-specific expression was apparent for *Loxl1* (GeneID: 16949), *Tll1* (GeneID: 21892), and *Pcolce2* (GeneID: 76477) (Figure 12C). Thus, in each prominence there will be a unique combination of collagens, collagen chaperones and processing enzymes that together will generate specific extracellular matrix environments.

#### Other ECM Components

A small number of genes encoding other structural proteins associated with the extracellular matrix were down-regulated in all prominences including alpha 1 laminin (*Lama1*, GeneID: 16772), *Smoc1* (GeneID: 64075), and *Matn3* (GeneID: 17182) (Figure 13A), or else displayed relatively level expression profiles (Figure 13B). However, many other genes encoding components of the nascent skeleton and extracellular matrix showed significant and coordinate expression increases in all prominences from E10.5–E12.5 (Figure 13C). Expression levels of genes encoding several laminins as well as other proteins involved in microfibril formation increased 2 to 6-fold. Similarly, transcripts of genes encoding proteoglycans, including decorin (*Dcn*, GeneID: 13179), biglycan (*Bgn*, GeneID: 12111), asporin (*Aspn*, GeneID: 66695), and osteoglycin (*Ogn*, GeneID: 18295), were also increasing up to 30-fold during this period. We note that some genes such as *Spon1* (GeneID: 233744) (Figure 13C) and *Emilin2* (GeneID: 246707) (Figure S5) exhibited more complex expression profiles, but were still coordinately regulated.

The coordinate regulation of genes encoding various ECM components was nevertheless associated with prominence-specific differences in the rate or degree of change over the time course of our analysis. Such differences are likely to yield some prominence-specific variation in extracellular matrix composition. More profound differences may originate from a subset of genes that displayed more distinctive prominence-specific expression patterns (Figures 13D). The MdP was distinguished by a dramatic increase in the expression levels of *Thbs4* (GeneID: 21828) compared with the other prominences, but relatively flat expression of *Sdc2* (GeneID: 15529). In the FNP, expression levels for *Tnn* (GeneID: 329278) remained stable whereas expression of this gene was changing in the MdP and MxP. In contrast, expression of *Acan* (GeneID: 11595), *Reln* (GeneID: 19699), and *Matn4* (GeneID: 17183), increased more in the FNP than in the other two prominences. Of note, *Matn4* expression rose ∼60-fold in the FNP over the time course of the analysis, compared with a 4-fold rise in the MxP. *Lad1* (GeneID: 16763) and *Hapln1* (GeneID: 12950) also showed FNP specific gene expression profiles.

In parallel with changes in expression of genes encoding structural components of the ECM, there were corresponding changes in transcript levels for those encoding modifying enzymes and inhibitors (Figure 14A–C). We did not observe decreases in the expression of such genes, and only a small subset displayed relatively constant transcript levels (Figure 14A). Instead, a majority of these genes including *Sulf1* (GeneID: 240725), *Mmp11* (GeneID: 17385), *Timp3* (GeneID: 21859), *Adam22* (GeneID: 11496), *Adamts20* (GeneID: 223838), and *Adamtsl1* (GeneID: 77739) showed a coordinate increase in expression in the three prominences (Figure 14B). Nevertheless, a number of genes had prominence-specific expression patterns, particularly *Fap* with a unique expression profile in each prominence (Figure 14C). In addition, rising levels of *Adam12* (GeneID: 11489) and *Adamts15* (GeneID: 235130) transcripts, as well as lower levels of *Has2* (GeneID: 15117) expression, distinguished the MdP from the MxP and FNP. The FNP itself had a different pattern of *Timp1* (GeneID: 21857), *Hs6st2* (GeneID: 50786), *Htra1* (GeneID: 56213), *Mmp2* (GeneID: 17390), and *Papss2* (GeneID: 23972) expression from the other two prominences. Overall, for all aspects of the ECM and its processing enzymes, the MxP showed fewer unique expression profiles than the MdP and FNP. Instead, it tended to have an intermediate profile, sharing some expression profiles with the FNP and others with the MdP.

### Genes Encoding Cell:Cell Communication and Cell Adhesion Molecules

The Gene Ontology and InterPro analyses shown in Figures 6 and 7 and Tables S3 and S4 highlighted gene categories associated with cell:cell adhesion as increasing in all prominences, and also suggested that claudin family members were differentially expressed in the FNP. Examination of individual genes associated with gap junctions, tight junctions, and desmosomes confirmed these dynamic and tissue-specific expression changes (Figure 15A–D). Genes encoding catenin proteins, which link the cytoskeleton with adherens junctions [34], showed coordinate decreasing (*Ctnnal1*, GeneID: 54366; Fig 15A), level (*Ctnna1*, GeneID: 12385; *Ctnnb1*, GeneID: 12387; and *Ctnnd1*, GeneID: 12388: Fig 15B), or increasing (*Ctnna2*, GeneID: 12386; Fig. 15C) expression profiles in the three prominences. Similarly, expression of the gap junction protein gene *Gjb3* (GeneID: 14620, connexin 31) was decreasing in all prominences, while there was a concomitant rise in *Gjb2* (GeneID: 14619, connexin 26) and *Gjb6* (GeneID: 14623, connexin 30) expression. Genes encoding desmosomal proteins, including *Dsc2* (GeneID: 13506), *Dsp* (GeneID: 109620), and *Perp* (GeneID: 64058), were also increasing in expression within in the MdP, MxP, and FNP between E10.5 and E12.5. These findings reflect increased adhesion, cell communication, and differentiation within epithelial sheets shared by the three facial prominences. Several genes associated with tight junctions, including *Tjp1* (GeneID: 21872), *Tjp2* (GeneID: 21873), and *Tjp3* (GeneID: 27375), had slightly decreasing or essentially level expression in the facial prominences. In contrast, the expression profiles for many other genes encoding adherens and tight junction proteins [35], [36] displayed markedly different dynamics in the FNP from the other two prominences (Figure 15D). With respect to adherens junctions, *Pkp2* (GeneID: 67451), *Ssx2ip* (GeneID: 99167) and *Shroom3* (GeneID: 27428) all showed higher expression in the FNP, as did the tight junction genes *Cgn* (GeneID: 70737) and *Ocln* (GeneID: 18260). Higher expression of the tight junction gene *Cldn1* (GeneID: 12737) was characteristic of the MdP and MxP, whereas *Cldn3* (GeneID: 12739), *Cldn6* (GeneID: 54419), *Cldn7* (GeneID: 53624), *Cldn9* (GeneID: 56863) were expressed at greater levels in the FNP throughout this developmental window. *Cldn11* (GeneID: 18417) and *Cldn23* (GeneID: 71908) expression also rose more rapidly in the FNP than in the other two prominences. Overall, these findings support a different composition and/or function of the junctional complexes in the FNP compared with the MdP and MxP. In this context, the regulatory molecule protein kinase C zeta (*Prkcz*, GeneID: 18762), which associates with tight junctions [35], [36], is also expressed at a higher level in the FNP. The MdP had higher levels of *Amot* (GeneID: 27494) expression, presumably associated with accelerated vasculature development in this prominence, as well as the cell:matrix adherens junction gene *Nexn* (GeneID: 68810).

The dynamic changes in the expression of genes encoding cell:cell junction components prompted a more detailed examination of genes involved in intercellular communication and cell adhesion (Figure 16A–D). A subset of these genes was coordinately down-regulated as development progressed (Figure 16A), especially *Igdcc3* (previously known as *Punc*, GeneID: 19289) whose expression decreased by 15 to 30-fold during our analysis. In agreement with our findings, previous reports showed that *Igdcc3*, which encodes a neural cell adhesion molecule, has widespread expression in the mesenchyme of the facial prominences at E10.5, but soon after becomes limited to discrete regions of the CNS [37], [38]. Several other genes had relatively stable expression levels (Figure 16B), including *Pvrl1* (GeneID: 58235), a gene that when mutated results in human CL/P [39], [40], [41]. However, consistent with the onset of differentiation, a majority of expression profiles were coordinately up-regulated in the facial prominences, including genes that encode specific cell adhesion molecules, cadherins, and protocadherins, which exhibited 2 to 10-fold increases (Figure 16C).

Prominence specific expression profiles were observed for several additional genes involved in cell adhesion (Figure 16D). In particular, in the MdP expression of *Cdh15* (GeneID: 12555), a gene associated with muscle differentiation [42], showed a sharp increase compared to the other prominences. Relatively low, stable, levels of *Pcdh8* (GeneID: 18530) and *Pcdh19* (GeneID: 279653) transcripts were also characteristic of the MdP. Distinctive expression profiles were not observed for the MxP, a finding in common with the data obtained from extracellular matrix gene analysis (see above). However, transcript levels for *Itga8* (GeneID: 241226) and *Itgb5* (GeneID: 16419) were slightly higher in the MxP compared to the other prominences. Several genes showed higher basal expression levels in the FNP, including those encoding the cell adhesion molecules *Pcdh20* (GeneID: 219257), *Pcdh21* (GeneID: 170677), *Cadm1* (GeneID: 54725), and *Epcam* (GeneID: 17075). In this context, *Pcdh21* is associated with olfactory function [43], and it is likely that some of the differences between the FNP and the two other prominences reflect the specialized function of the olfactory epithelium.

### Transcription Factors

Although we observed significant changes in the expression of genes involved in cell cycle, cell adhesion, cell structure, and the extracellular matrix, by far the most dramatic molecular signatures of the prominences were provided by genes encoding sequence-specific DNA binding transcription factors (Figures 17A–D, 18A–C). Multiple transcription factors displayed coordinated gene expression in the three prominences over the time course of this analysis (Figure 17A–C and Figure S6). A small number of genes were down-regulated (Figures 8A and 17A) including those encoding p53 (GeneID: 22059), E2F5 (GeneID: 13559), and Sp6 (GeneID: 83395) whose expression dropped 2 to 4-fold correlating with the reduction in cell proliferation and general cellular metabolism noted above. Transcription factors implicated in maintenance of stem cell function, including *Sall4* (GeneID: 99377) and *Zbtb16* (*Plzf*, GeneID: 235320) [44], [45], [46], or in the expansion of precursor populations, such as *Arid3b* (GeneID: 56380) [*47*], were also down-regulated ∼5-fold. Several genes were expressed at relatively stable levels in the three prominences, including members of the Stat and Smad transcription factor families and *Cited2* (GeneID: 17684), which encodes a transcriptional modulator (Figure 17B). The expression of a much larger group of genes was up-regulated in all prominences (Figure 17C). This class of genes includes *Fosl2* (GeneID: 14284), *Runx2* (GeneID: 12393), and *Sp7* (*osterix*, GeneID: 170574), which are required for development of skeletal elements [48], [49], [50], and whose transcript levels rose 5 to 15-fold during this developmental window. Similarly, expression of *Klf4* (GeneID: 16600), a gene responsible for skin barrier function in the differentiating epidermis [51], was increased ∼4 to 8-fold in all three prominences. *Irf6* (GeneID: 54139), a gene expressed in facial ectoderm and critical for patterning mouse orofacial development as well as mutated in human CL/P [52], [53], [54], was also present in this category. Several genes that encode components of signal transduction pathways in many cell types, including *Stat3* (GeneID: 20848), and the nuclear hormone receptors, corepressors, and coactivators *Ncoa1* (GeneID: 17977), *Ncor2* (GeneID: 20602), *Nr3c1* (GeneID: 14815) and *Thra* (GeneID: 21833), were also coordinately up-regulated. In addition, all members of the NFI transcription factor gene family demonstrated coordinate 10 to 50-fold increases in expression over this time period in the facial prominences. A further group of transcription factor genes displayed distinctive patterns of expression in each of the three prominences that did not fall into the simple categories noted above (Figure 17D). This group was quite small and included genes such as *Sox8* (GeneID: 20681) and *Irx4* (GeneID: 50916), which had transcript levels rising and falling in different prominences. Another example was *Foxg1* (GeneID: 15228), with stable expression in each prominence, but with a low, intermediate, and high level of expression in the MdP, MxP and FNP respectively.

The microarray data were also processed to ascertain those transcription factor genes that exhibited prominence-specific expression or were coordinately regulated in any two of the three prominences (Figure 18, and Figures S7 and S8). This analysis revealed a complex series of expression profiles for each prominence, with the MdP and FNP showing the greatest differences. With respect to the MdP, our analysis showed that several genes required for muscle differentiation [29] including *Tcf21* (*capsulin*, GeneID: 21412), *Myf5* (GeneID: 17877), *Myod1* (GeneID: 17927), *Myog* (GeneID: 17928) and *Msc* (GeneID: 17681), were expressed at higher levels in this prominence (Figure 18A). Expression of *Tcf21* was essentially level throughout the time course, with levels 2 to 3-fold higher than in the MxP and FNP. Expression of the other genes increased between 4 to 30-fold between E10.5–E12.5, presumably reflecting development of the tongue and the muscles of mastication [55]. Additional genes more highly expressed in the MdP included *Dlx5*, *Dlx6*, *Gsc*, *Hand1* (GeneID: 15110), and *Hand2*, consistent with their developmental roles in patterning the mandible [24], [26].

The MxP shared multiple expression profiles with either the MdP or FNP, and only a few genes showed expression differences specific to this prominence – and most of these were subtle (Figure 18B). Thus, *Foxc2* (GeneID: 14234) and *Pknox2* (GeneID: 208076) showed a slight decrease in expression in the MxP compared with the other prominences, whereas levels of *Foxl2* (GeneID: 26927), *Irx3* (GeneID: 16373) and *Irx5* (GeneID: 54352) were slightly higher. The FNP displayed higher expression for genes encoding transcription factors involved in specification and development of ectodermal placodes and in neurogenesis [56], [57], including *Pax6* (GeneID: 18508), *Sox2* (GeneID: 20674), *Ascl1* (GeneID: 17172), *Olig1* (GeneID: 50914) and *Olig2* (GeneID: 50913) (Figure 18C). This observation again presumably reflects the development of the olfactory placodes and specialized olfactory epithelium in the FNP. Transcripts of genes involved in patterning the FNP, such as *Pax7* (GeneID: 18509), were also expressed at a higher level in this prominence (Figures 17D). The integration of the multiple differences in transcription factor gene expression profiles is likely to provide a powerful driving force for discriminating the different prominences during development.

### Genes Associated with Chromatin Dynamics

We also examined genes associated with chromatin structure (Figure 19A–D) since alterations in DNA packaging would be expected to accompany the rapidly changing profiles of transcription factors, as well as the switch between genes required for cell cycle progression versus differentiation. As indicated by the trajectory clustering analysis (Table S2), we observed dynamic changes in expression of genes associated with chromatin remodeling, DNA methylation, and gene regulation. Our findings imply that there is a switch from a progenitor to a differentiated cell program of gene accessibility during the period under analysis in all prominences. Thus, expression of genes encoding particular members of the Chd family of chromatin remodeling proteins was decreasing (*Chd1*, GeneID: 12648; *Chd7* GeneID: 320790; Figure 19A), while others remained relatively constant (*Chd8*, GeneID: 67772; Figure 19B), and at least one was increasing (*Chd9*, GeneID: 109151; Figure 19C). These findings may reflect the difference between maintaining chromatin assembly functions and epigenetic marks during DNA replication versus the established role of *Chd9* in regulating genes required for skeletal tissue development [58]. An alteration in chromatin dynamics was also indicated by the increasing expression of genes encoding many components of the SWI/SNF remodeling complex, including *Smarca1* (GeneID: 93761), *Smarca2* (GeneID: 67155), *Smarcc2* (GeneID: 68094) and *Smarcd3* (GeneID: 66993) (Figure 19C), whereas others such as *Smarcc1* (GeneID: 20588) and *Smarce1* (GeneID: 57376) showed relatively constant expression levels (Figure 19B). The majority of these chromatin remodeling genes showed coordinate regulation of expression, with the exception of *Baz1a* (GeneID: 217578), which was preferentially down-regulated in the MdP (Figure 19D). There was also a general increase in transcripts for genes involved in histone acetylation, including *Kat2b* (*Pcaf*, GeneID: 18519) and *Myst4* (GeneID: 54169), suggesting an overall increase in chromatin accessibility. Simultaneously, expression of genes encoding specific components of the histone deacetylase and methyltransferase machinery declined over this developmental window, including *Sap18* (GeneID: 20220), *Sap30* (GeneID: 60406), and *Suv39h2* (GeneID: 64707). Interestingly, though, transcripts for the HDAC components *Hdac5* (GeneID: 15184) and *Chd3* (GeneID: 216848) were rising. There was also a 2-fold increase in transcripts for the deacetylase *Phf21a* (GeneID: 192285), which may reflect the importance of this gene in the repression of neural genes in non-neural tissues [59].

With respect to polycomb group and trithorax genes, expression of *Asxl3* (GeneID: 211961), *Cbx4* (GeneID: 12418), *Cbx6* (GeneID: 494448) and *Mll5* (GeneID: 69188) increased during orofacial development (Figure 19C). These genes encode the archetypal epigenetic marker proteins responsible for repression of inappropriate genes in differentiated cells [60]. In contrast, transcripts corresponding to *Pcgf6* (GeneID: 71041), *Cbx1* (GeneID: 12412), and *Cbx2* (GeneID: 12416) decreased between E10.5–E12.5 in the facial prominences. These data imply that such polycomb proteins could be responsible for maintaining progenitor cells by repression of genes required for commitment to differentiation. *Dnmt3b* (GeneID: 13436), encoding an enzyme involved in *de novo* CpG DNA methylation, which is associated with transcriptional repression and heterochromatin formation [61], was also down-regulated between E10.5–E12.5 (Figure 19A). In contrast, *Dnmt3a* (GeneID: 13435) showed flat or increasing expression depending on the probe used (data not shown). The timing of the switch between these related DNA methylases, also observed in embryos at the protein level [62], potentially indicates that they have different roles in early progenitor versus differentiated cells. The dynamics of *Dnmt3b* expression are also of interest given its mutation in human immunodeficiency-centromeric instability-facial anomalies (IFC) syndrome (OMIM: 242860) [61]. Taken together, the changes in the expression of genes involved in DNA modification and chromatin structure demonstrate that the genome is undergoing rapid and dynamic changes that will greatly alter transcription factor accessibility.

### Analysis of Novel Facial Gene Expression

**Figure 20:**
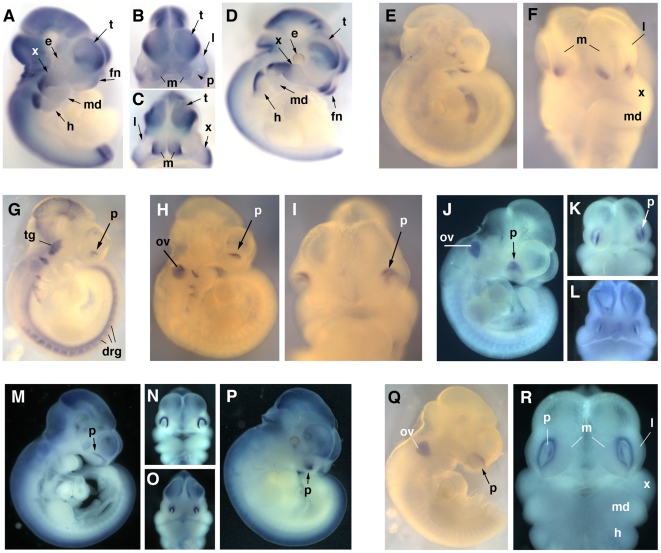
Expression of representative genes in the facial prominences I: global and FNP-specific genes. Whole mount in situ expression analysis of: *2610017I09Rik* (A–D); *9430073N08Rik*, *Fam162b* (E, F); *Elav14* [*HuD*] (G); *Prr15* (H, I); *Fam107a* (J–L); *Wscd1* (M–P); and *Image 6309403* (Q, R) showing lateral (A, D, E, G, H, J, M, P, Q) or ventral (B, C, F, I, K, L, N, O, R) views of E10.5 (A, B, E–K, M, N, Q, R) or E11.5 (C, D, L, O, P) mice. Note for panel G that despite the clear expression of *Elavl4* in the trigeminal ganglia, we did not detect expression in the MxP and MdP RNA samples we had purified for microarray analysis. This finding provides additional proof that the samples we have purified were not significantly contaminated with surrounding tissue. Abbreviations: drg, dorsal root ganglia; e, eye; fn, frontonasal prominence; h, hyoid arch; l, lateral nasal prominence; m, medial nasal prominence; md, mandibular prominence; ov, otic vesicle; p, nasal pit; t, telencephalon; tg, trigeminal ganglion; x, maxillary prominence.

**Figure 21:**
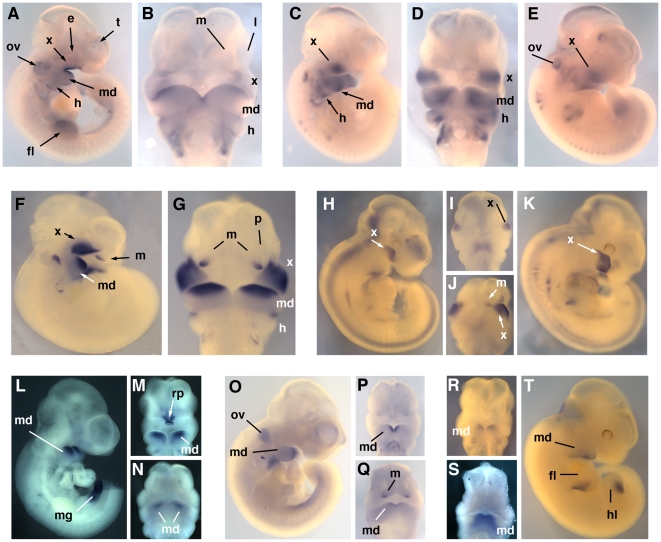
Expression of representative genes in the facial prominences II: MdP and MxP-specific. Whole mount in situ expression analysis of: *Ifitm3* (A, B); *Lrba* (C–E); *AI606473* (F, G); *A930038C07Rik* (H–K); *Gpr50* (L–N); *Rgs5* (O–Q); and *AV026068* (R–T); showing lateral (A, C, E, F, H, K, L, O, T) or ventral (B, D, G, I, J, M, N, P–S) views of E10.5 (A–D, F–I, L, M, O, P, R) or E11.5 (E, J, K, N, Q, S, T) mice. Abbreviations: e, eye; fl, forelimb bud; fn, frontonasal prominence; h, hyoid arch; hl, hindlimb bud; l, lateral nasal prominence; m, medial nasal prominence; md, mandibular prominence; mg, midgut; ov, otic vesicle; p, nasal pit; rp, Rathke's pouch; t, telencephalon; x, maxillary prominence.

Our studies highlighted a large number of genes not previously associated with the developing face. This group of genes included well-studied genes as well as novel genes for which little or no functional information exists. We selected a number of these genes for further analysis based, in part, upon their high levels of expression in discrete prominences as determined by the microarray analysis (Figure S9). RNA *in situ* probes were designed for each gene and WMISH was employed to determine the location of expression within each prominence between E10.5–E12.5. The spatial distribution of transcripts observed using WMISH displayed excellent agreement with the microarray expression results, providing additional verification of our dataset (Figures 20, 21, and Figure S9). Figure 20A–D shows the WMISH data obtained for cDNA *2610017I09Rik* (GeneID: 66297), a transcript of unknown function [63], that was readily detected by microarray analysis in each prominence at all time points studied. WMISH confirmed that this gene was expressed highly in all three prominences, but also indicated that the distribution was not uniform. Expression of *261007I09Rik* showed a proximal distribution in the MdP and MxP, whereas in the FNP it was more concentrated in the medial rather than lateral nasal prominences. *2610017I09Rik* maps to mouse chromosome 1, in close vicinity to *Pou3f3* (GeneID: 18993) (Figure S10), with which it shares a similar expression profile in the facial prominences and CNS.

The additional transcripts highlighted in Figure 20 show preferential expression in the FNP over the other prominences, in agreement with the microarray data. One of these, corresponding to the cDNA *9430073N08Rik* (GeneID: 77296), was expressed in a portion of both the lateral and medial components of the nasal prominence proximal to the oral cavity (Figure 20E, F). This latter gene, *Fam162b*, is predicted to encode an integral membrane protein, but little else is know about its function [63]. For the rest of the genes that display FNP-specific expression, we utilized the distribution of *Elavl4* (*Hud*) transcripts as a landmark ([27]; Figure 20G). Expression of *Elavl4* was elevated in the FNP and localized to the developing olfactory epithelium of the nasal pit consistent with its association with neurogenesis. The other FNP-enriched transcripts analyzed (*Prr15*, GeneID: 78004; *Fam107a*, GeneID: 268709; *Wscd1*, GeneID: 216881; and *Image:6309403*, GenBank gi: 21854763) were similarly localized in the nasal pit (Figure 20H–R), presumably reflecting a role for these genes in olfactory neurogenesis. Expression of *Prr15* (also termed *G90*), which may encode a proline-rich protein or act as a non-coding RNA, has previously been observed in post-mitotic cells, including within the olfactory epithelium ([64]; Figure 20H, I). *Fam107a* (Figure 20J–L) (also known as *BC055107, DRR1* and *TU3A*) may encode a nuclear protein linked to growth control and tumorigenesis [65], [66]. *Wscd1* (Figure 20, M–P) is a wsc domain-containing protein predicted to be integral to the cell membrane [63]. The *Image:6309403* transcript (Figure 20Q, R) is predicted to encode microRNA 429 (*mir429*, GeneID: 723865) of unknown function [63].

WMISH was also used to examine genes that showed high expression in the MdP and/or MxP in the microarray analysis, and once again we observed good agreement between the data obtained using the two methods (Figure 21 and Figure S9). *Ifitm3* (GeneID: 66141), *Lrba* (GeneID: 80877), and *AI606473* (GeneID: 99686) were expressed in both the MdP and MxP, although with clearly different spatiotemporal distribution patterns. *Ifitm3* (interferon induced transmembrane protein 3) [67] was expressed primarily in the ectoderm of the cleft between the MdP and MxP (Figure 21A, B). *Lrba*, which encodes an LPS-responsive beige-like anchor protein that may be involved in vesicle trafficking and Notch regulation [68], was expressed more diffusely in the MdP and MxP as well as in the more caudal branchial arches (Figure 21C–E). Expression of *AI606473*, a gene of unknown function [63], occurred primarily in the MxP and the distal portion of the MdP, although a discrete domain of expression was also seen in the medial nasal prominences (Figure 21F, G). This expression pattern showed strong similarity with that of *Lhx8* ([25]; Figure S9). *AI606473* is located just upstream of *Lhx8* on mouse chromosome 3 and is transcribed in the opposite direction (Figure S10), suggesting that these two genes may share common *cis*-regulatory elements and/or a bidirectional promoter.

Other genes analyzed displayed more prominence-specific gene expression patterns. *A930038C07Rik* (GeneID: 68169) showed the highest levels of expression in the MxP, particularly between E11–E12, and serves as a valuable new marker for this prominence (Figure 21, H–K). This gene, known as *C4orf31* (GeneID: 79625) in human, encodes a hypothetical membrane protein and is negatively regulated by *Hox* genes in the developing kidney [69]. This latter observation is intriguing given that the anterior-posterior Hox code terminates just caudal to the MxP [70]. Specific expression in the MdP was seen for two genes linked with G protein coupled signaling [71], [72], *Gpr50* (GeneID: 14765) (Figure 21, L–N) and *Rgs5* (GeneID: 19737) (Figure 21, O–Q). Expression of *Gpr50* was concentrated in the rostral MdP, whereas *Rgs5* was expressed more distally. At E10.5, both genes were expressed at high levels, but expression decreased considerably from E11.5 onwards. Expression of *Gpr50*, a melatonin-related orphan receptor, was also associated with Rathke's pouch in the roof of the oral cavity, suggesting a role in the development of the pituitary gland. *AV026068* (GeneID: 102265), termed *NBLA00301* in human (GeneID: 79804), encodes a short hypothetical protein of unknown function [63], and its transcripts were expressed at elevated levels in the MdP throughout the developmental time course (Figure 21, R–T). The expression domain of *AV026068* in the distal MdP, as well as in the posterior region of the limb bud, resembled that of the aforementioned *Hand2* gene (Figure 4). *AV026068* is positioned adjacent to *Hand2* on mouse chromosome 8, but is expressed from the opposite strand beginning several kb upstream of the latter's transcriptional start site (Figure S10). This arrangement is remarkably similar to those described above for *2610017I09Rik* and *Pou3f3* as well as *Lhx8* and *AI606473*. Again, the similarity between the expression patterns of *AV026068* and *Hand2*, as well as the bidirectional nature of these two gene's transcription units, suggests that they share common mandible-specific *cis*-regulatory sequences. The finding that this arrangement occurs for these three new pairs of genes indicates that it may be a common mechanism of coordinating gene expression in the developing mouse face.

## Discussion

The development of the vertebrate face is an intricate process that requires the coordinate growth, morphogenesis, and fusion of separate prominences [2], [3], [4]. Although the face eventually forms an integrated structure, the paired mouse facial prominences – the mandibular, maxillary, and frontonasal – are fated to form distinct functional components of the skeleton, the sensory system, and the respiratory and digestive systems. Variations in the growth properties and derivatives of these prominences have been a major factor driving vertebrate evolution and the expansion of vertebrate species into disparate ecological niches. Furthermore, aberrant development of these prominences is a major factor in human orofacial clefting and other craniofacial birth defects.

Although previous studies have identified many individual genes and pathways required for facial development, we still lack a clear understanding of the gene networks regulating face formation [2]. Here we have utilized a systems biology approach to document the panoply of genes expressed during a critical period of mouse orofacial development. The genome-wide analysis of transcripts present in the individual facial prominences yields a comprehensive overview of the similarities and differences in gene expression among the three facial prominences at five time points over a two day developmental window. We have confirmed individual aspects of our microarray expression dataset by comparing our results with known expression patterns of representative genes, as well as by WMISH studies of selected novel orofacial transcripts. This provides a robust and powerful dataset with low variance due to a combination of stringent experimental protocols and multiple biological replicates and its statistical strengths make possible sophisticated bioinformatic analyses and network predictions [55], [73]. On a more basic level, the dataset may help uncover individual genes or groups of genes that have previously been overlooked as components of the repertoire required for face formation or which can be reactivated in head and neck cancer.

A central component of our present analysis has been to identify stage- and tissue-specific gene signatures associated with the developing facial prominences. We determined that the facial tissue we analyzed contained >20,000 expressed transcripts, many of which were coordinately regulated in the three prominences. Overall, there is a general decrease in the expression of genes involved with basic growth and metabolic processes in all prominences between E10.5–E12.5. There is also down-regulation of genes that are associated with the maintenance of progenitor cell function, including *Igdcc3* (*Punc*), *Sall4*, a subset of polycomb genes, and certain DNA and chromatin modifying components. Balanced against the down-regulation of genes involved in growth, replication, and progenitor cell function, there is corresponding up-regulation of transcripts derived from genes involved in differentiation in the MdP, MxP and FNP. These coordinated changes in gene expression in the three prominences are accompanied by other unique patterns of gene expression in each location that presage the distinct tissues generated by each prominence. Thus, the FNP is distinguished by the expression of genes associated with development of the olfactory placodes and neurogenesis in the olfactory epithelium. In contrast, transcripts from genes involved in vasculogenesis and muscle development are prominent in the MdP correlating with the development of the branchial arch arteries and tongue from the first arch [3], [4], [55].

**Figure 22:**
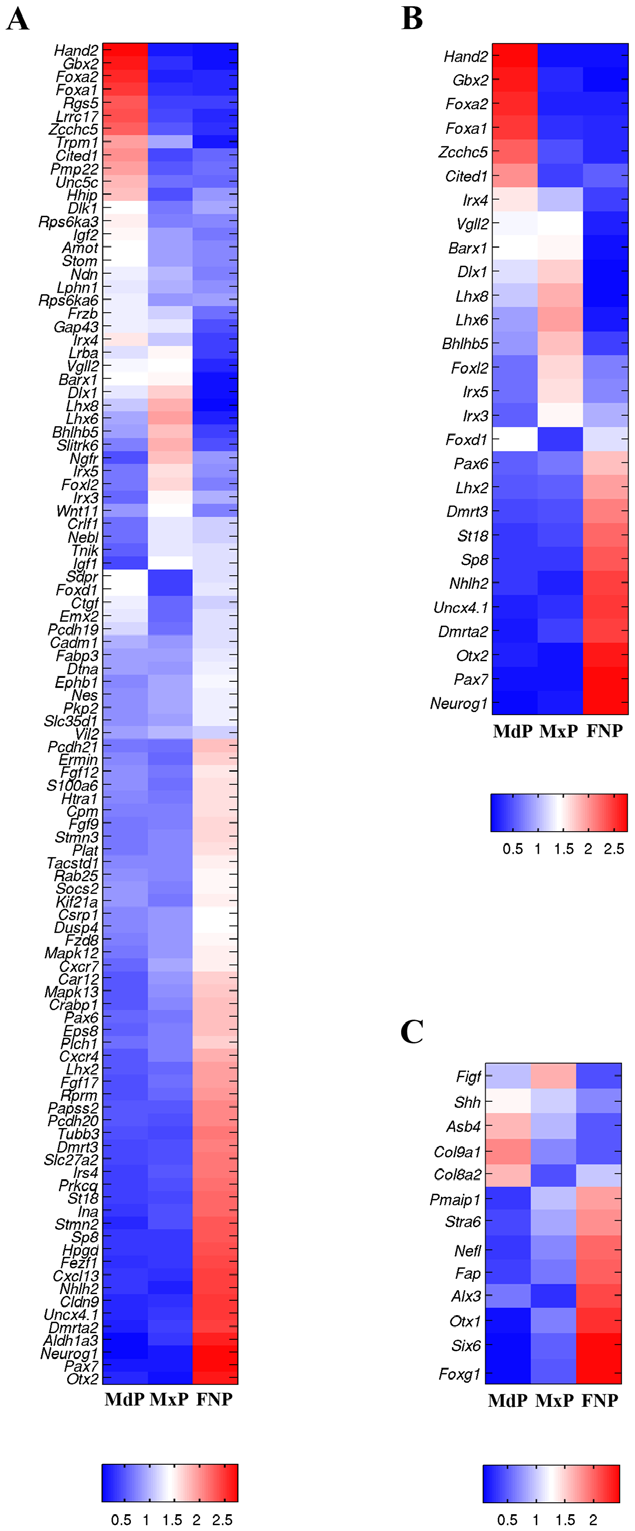
Significant expression differences exist among the facial prominences at E10.5. Heatmaps of (A) genes showing ∼2-fold or greater differences in expression in the three prominences at E10.5; (B) the transcription factors within this dataset; (C) genes showing graded expression in the three prominences. The genes analyzed are shown on the left and the prominences at the bottom. For each gene, the data was first averaged over all seven replicates at E10.5 for each probe set on the array and then averaged across all probe sets representing the gene, where probe sets called ‘Absent’ across all 105 arrays were previously discarded. The red-blue color of the heatmap indicates the range of the resulting data for each gene, scaled relative to the mean of the three values at E10.5 in the three prominences.

Fundamental differences in gene expression between the three prominences are already apparent at E10.5, the earliest time point analyzed. Thus, early in facial development, unique molecular programs are employed in each prominence that presumably drive the different fates of these three structures. The most robust prominence-specific expression differences occur in the MdP and FNP, with a more limited number of expression profiles that are distinctive for the MxP (see Figure 22A, B). The FNP, in particular, shows the most distinctive gene expression profile with increased expression of many genes involved in transcription (e.g. *Dmrta2*, GeneID: 242620; and *Dmrt3*, GeneID: 240590), signaling (e.g. *Cxcl13*, GeneID: 55985; *Cxcr4*, GeneID: 12767; *Fezf1*, GeneID: 73191; *Irs4*, GeneID: 16370; and *S100a6*, GeneID: 20200) and also retinoid metabolism (*Aldh1a3*, GeneID: 56847). Conversely, compared with the other prominences, the FNP has reduced RNA levels for several transcription factors (e.g. *Barx1* GeneID: 12022; and V*gll2*, GeneID: 215031) as well as *Frzb* (GeneID: 20378), *Lrba* and *Gap43* (GeneID: 14432). The MdP and MxP also have a unique arrangement of genes that are either expressed at higher or lower levels in either of these individual prominences. We note that another group of genes exhibit a unique level of expression in each of the prominences, possibly reflecting a spatial code of graded expression that helps to impart identity (Figure 22C).

Overall, genes encoding transcription factors and molecules involved in signal transduction figure heavily in prominence-specific differences at the onset of facial outgrowth. Considered together, the combinatorial repertoire of transcription factors present in each prominence by E10.5 will certainly generate unique and highly specific patterns of gene expression. Moreover, each prominence is primed to express different signal transduction components and will therefore respond differently to any available signaling molecules. Together, these differences undoubtedly provide the basis for the elaboration of individual FNP, MxP, and MdP identities as development proceeds. Given that fundamental differences in gene expression in these prominences are already apparent by E10.5, it will be important to extend these studies to even earlier time points to determine when specific patterns are established. However, the extremely small size of tissue samples that can be prepared from the FNP and MxP prior to E10.5 would provide lower quality bioinformatic information than presented in the current analysis.

The data we have obtained can be employed to predict and interpret regulatory networks, nodal points, and gene interactions shaping the mammalian face [55], [73]. One unexpected finding from our analyses was that several genes expressed in the developing face are organized into bidirectional transcription units (Figure S10). Such paired genes have similar expression patterns, presumably due to the presence of shared *cis*-regulatory sequences. Recently, a bidirectional transcription unit was noted for another gene-pair expressed in the face, *Foxl2* and *E330015D05* (GenBank gi: 74228500), as well as for one of the pairs we describe *Pou3f3* and *2610017I09Rik* [74]. In all these instances the gene pairs are unrelated at the sequence level and are transcribed divergently. Nevertheless, the close linkage of two genes expressed in similar domains during facial development is reminiscent of the paired, although convergently transcribed, *Dlx1/2*, *Dlx3/4*, and *Dlx5/6* genes [26]. These new findings raise the possibility that such paired and/or bidirectional transcription units may be a more common feature of gene expression in the face than was previously appreciated. At present, the functional consequence of this arrangement for many of these gene-pairs remains unclear, but the possibility that targeted mutation of one gene could affect expression or function of the other partner in the transcription unit should be considered.

Recent studies have revealed links between facial morphology and the action of multiple signal transduction pathways including those involving Calcium ion, Fgf, Shh, BMP, and Wnt signals [2], [75]. Mining this large expression dataset should assist in identifying associated components of these signaling pathways that function in orofacial formation. On one level, individual genes can be prioritized for analysis by gene knockdown in animal model systems, but we also hope that the dataset can be utilized to revisit previously generated gene knockouts in mice to screen for subtle defects in orofacial formation and function that may not have been recorded. Further, the available information may help to predict combinations of genes that need to be targeted simultaneously to impact orofacial development due to redundancy. Conversely, it may be possible to generate homeotic transformations by altering key gene expression patterns in one prominence to match a different facial region. In a wider context, the data can also be utilized in conjunction with microarray analyses performed on orofacial development in other species, particularly human and chick, to study species-specific differences in gene expression that may underlie variation in vertebrate facial morphology [76], [77]. Finally, the information we have obtained can also be utilized as a baseline dataset that can be employed to identify gene expression differences that occur in various mouse models of orofacial clefting or other types of craniofacial dysmorphology.

## Supporting Information

Figure S1 Assessment of RNA quality prior to microarray analysis. A representative RT-PCR analysis on RNA isolated from the FNP and MxP for four genes expected to be expressed in the facial prominences (Tcfap2a, Bmp2, Dlx2, and Gsc). The sizes of the expected products are shown next to the gene name. Zic3 is expressed in forebrain tissue and its absence from the facial prominence RNA samples following RT-PCR and gel electrophoresis was indicative of lack of contamination by CNS tissue. The intact mouse head sample serves as a positive control for all RT-PCR reactions. MKS, DNA ladder size markers.
(1.31 MB TIF)

Figure S2 Spatial and Temporal Specific Gene Expression Differences in the Developing Facial Prominences. A. A bar graph illustrating the number of probes showing significant differences between the two prominences indicated at a given time point assessed using limma based on a 1% false discovery rate and a 2-fold expression difference. B. Bar graph showing the number of probe sets showing a significant increase or decrease between adjacent time points within a particular prominence using the same statistical criteria employed in A.
(0.56 MB TIF)

Figure S3 Analysis of Signal values among replicates. A–B. Boxplot of logarithm of mean and standard deviation of the Signal values among seven replicates. The horizontal axis shows the sample index 1–15 corresponding to time points E10.5 to E12.5 in MdP (1–5), MxP (6–10) and FNP (11–15) respectively. Boxes have lines at the lower quartile, median and upper quartile values while whiskers extending from the box show the remaining data range. The consistency of box size and median level across all boxes shows reproducibility among replicates in all prominences and at all time points. C–D. Worst (C) and best (D) replication among a pair of replicates. Each dot represents the Signal value for a probe. The identity of the sample is given along the axes and coefficient r measures linear correlation. The wide and slim spread of points in each plot demonstrates high and low quality replication, respectively.
(0.37 MB TIF)

Figure S4 Heatmap of collagen and collagen processing enzyme gene expression. The genes analyzed are shown on the left, the prominences at the top, the time points at the bottom and the class of profile at the right. The grey line marks the separation of up-regulated collagen genes from processing enzymes. Data for a given gene is the average of data for all probe sets representing that gene (first averaged within the seven biological replicates per time point) and then the resulting vector is scaled to have a mean of zero and a magnitude of one. Blue and red indicate low and high expression levels, respectively.
(0.87 MB TIF)

Figure S5 Heatmap of extracellular matrix component gene expression. The genes analyzed are shown on the left, the prominences at the top, the time points at the bottom and the class of profile at the right. Data for a given gene is the average of data for all probe sets representing that gene (first averaged within the seven biological replicates per time point) and then the resulting vector is scaled to have a mean of zero and a magnitude of one. Blue and red indicate low and high expression levels, respectively. VAR, variable profile but still coordinately regulated.
(1.04 MB TIF)

Figure S6 Heatmap of transcription factors coordinately regulated in all prominences. The genes analyzed are shown on the left, the prominences at the top, the time points at the bottom and the class of profile at the right. Data for a given gene is the average of data for all probe sets representing that gene (first averaged within the seven biological replicates per time point) and then the resulting vector is scaled to have a mean of zero and a magnitude of one. Blue and red indicate low and high expression levels, respectively. VAR, variable profile but still coordinately regulated.
(0.59 MB TIF)

Figure S7 Heatmap of transcription factors highly expressed in a single prominence. The genes analyzed are shown on the left, the prominences at the top, the time points at the bottom and the class of profile at the right. Data for a given gene is the average of data for all probe sets representing that gene (first averaged within the seven biological replicates per time point) and then the resulting vector is scaled to have a mean of zero and a magnitude of one. Blue and red indicate low and high expression levels, respectively. HIGH, expression is generally higher throughout the time course than in the other prominences, although not necessarily increasing or decreasing.
(3.14 MB TIF)

Figure S8 Heatmap of transcription factors highly expressed in two of the three prominences. The genes analyzed are shown on the left, the prominences at the top, the time points at the bottom and the class of profile at the right. Data for a given gene is the average of data for all probe sets representing that gene (first averaged within the seven biological replicates per time point) and then the resulting vector is scaled to have a mean of zero and a magnitude of one. Blue and red indicate low and high expression levels, respectively. HIGH, expression is generally higher throughout the time course in two of the three prominences, although not necessarily increasing or decreasing.
(2.68 MB TIF)

Figure S9 (A). Biological verification of the dataset using the novel genes associated with orofacial expression as well as the previously characterized linked genes. Raw data for the genes and associated probe sets (left) indicated at the five time points in the three prominences. (B). Corresponding heatmap showing the expression data for each probe scaled such that the vector of log2 expression values for a probe (averaged among replicate samples per time point) has a mean of zero and a magnitude of one. Red and blue indicate high and low expression, respectively.
(1.16 MB TIF)

Figure S10 Bidirectional transcription units associated with orofacial gene expression. The analysis of novel genes associated with expression in the facial prominences indicated that at least three mouse genes displaying similar expression patterns to known genes were also closely linked to them in the mouse genome and transcribed in the opposite direction. These finding suggest that they share common cis-acting sequences responsible for their similar patterns of gene expression. The distances between the pairs 2610017I09Rik and Pou3f3, AI606473 and Lhx8, and AV026068 and Hand2 are approximately 2.3 kb, 260 bp and 60–220 bp respectively. A bidirectional arrangement of these gene pairs is also conserved in the human genome (human cDNA or gene names are shown in blue), with the following caveats. The AK096498 (Homo sapiens cDNA FLJ39179 fis, clone OCBBF2004147) mRNA initiates ∼4 kb upstream of POU3F3, but other spliced transcripts from this locus begin only ∼2.3 kb upstream. The human RefSeq for LHX8 contains two upstream exons that are not present in the mouse RefSeq. The opposite strand transcript from AK055631 would therefore initiate from within the second intron of LHX8. However, we suspect (based on a protein sequence comparison between mammalian species) that the human RefSeq may have been derived from an atypical transcript and that the arrangement we have noted in the mouse may also hold in the human genome. Information was obtained from NCBI (http://www.ncbi.nlm.nih.gov/) and the UCSC genome browser (http://genome.ucsc.edu/) using the Mouse July 2007 (mm9) Assembly and the Human Feb. 2009 (hg19) Assembly.
(0.03 MB DOC)

Table S1 Gene specific PCR primer sequences and PCR product sizes.
(0.26 MB DOC)

Table S2 Trajectory Clustering Analysis. Trajectory clustering was applied using the limma-derived p-values with fdr = 0.01. Each line lists a specific over-represented GO Biological Process, Molecular Function, or InterPro term and the trajectory clusters within each prominence in which that term was over-represented. The trajectory profile is given as a sequence of ‘\’ for Decrease, ‘-’for Flat, and ‘/’ for Increase between consecutive time points. The prominence is specified as mandibular (MdP), maxillary (MxP) and frontonasal (FNP). Over-representation was tested using the binomial distribution, applying the Benjamini and Hochberg (6) false discovery rate multiple comparison correction at a cutoff of 0.05.
(0.06 MB XLS)

Table S3 Functional Category Analysis A–D. Details of Functional Category Analysis for Predominantly Decreasing Trajectories (With 2-Fold Change Requirement). Using the Trajectory Clustering Algorithm with limma-derived p-values and an fdr of 0.01 (2-fold change requirement), the combined set of all MGI identifiers represented by probes in clusters with at least two decreases and no increases was tested for over-representation of annotations. For each term, values used in calculating the binomial distribution are given as follows for each of the three prominences (P for mandibular (MdP), X for maxillary (MxP) and N for frontonasal (FNP)): H - whether the category was called significant at the (unadjusted) 0.05 level with the binomial distribution, N - number of MGI identifiers annotated to any category over the set of all MGI identifiers, M - number of MGI identifiers annotated to the given category, K - number of MGI identifiers tested in the list, and x - number of MGI identifiers in the list annotated to the given category. Annotations for (A) GO Biological Process (GOBP); (B) GO Molecular Function (GOMF); (C) InterPro; and (D) KEGG. E–J. Details of Functional Category Analysis for Predominantly Decreasing Trajectories (No Fold Change Requirement). Using the Trajectory Clustering Algorithm with limma-derived p-values and an fdr of 0.01 (no fold change requirement), the combined set of all MGI identifiers represented by probes in clusters with at least two decreases and no increases was tested for over-representation of annotations. For each term, values used in calculating the binomial distribution are given as follows for each of the three prominences (P for mandibular (MdP), X for maxillary (MxP) and N for frontonasal (FNP)): H - whether the category was called significant at the (unadjusted) 0.05 level with the binomial distribution, N - number of MGI identifiers annotated to any category over the set of all MGI identifiers, M - number of MGI identifiers annotated to the given category, K - number of MGI identifiers tested in the list, and x - number of MGI identifiers in the list annotated to the given category. Annotations for (E) GO Biological Process (GOBP); (F) GO Molecular Function (GOMF); (G) InterPro; (H) KEGG; and (J) MGI Phenotype. K–P. Details of Functional Category Analysis for Predominantly Increasing Trajectories (With 2-Fold Change Requirement). Using the Trajectory Clustering Algorithm with limma-derived p-values and an fdr of 0.01 (2-fold change requirement), the combined set of all MGI identifiers represented by probes in clusters with at least two increases and no decreases was tested for over-representation of annotations. For each term, values used in calculating the binomial distribution are given as follows for each of the three prominences (P for mandibular (MdP), X for maxillary (MxP) and N for frontonasal (FNP)): H - whether the category was called significant at the (unadjusted) 0.05 level with the binomial distribution, N - number of MGI identifiers annotated to any category over the set of all MGI identifiers, M - number of MGI identifiers annotated to the given category, K - number of MGI identifiers tested in the list, and x - number of MGI identifiers in the list annotated to the given category. Annotations for (K) GO Biological Process (GOBP); (L) GO Molecular Function (GOMF); (M) InterPro; (N) KEGG; and (P) MGI Phenotype. Q–U. Details of Functional Category Analysis for Predominantly Increasing Trajectories (No Fold Change Requirement). Using the Trajectory Clustering Algorithm with limma-derived p-values and an fdr of 0.01 (no fold change requirement), the combined set of all MGI identifiers represented by probes in clusters with at least two increases and no decreases was tested for over-representation of annotations. For each term, values used in calculating the binomial distribution are given as follows for each of the three prominences (P for mandibular (MdP), X for maxillary (MxP) and N for frontonasal (FNP)): H - whether the category was called significant at the (unadjusted) 0.05 level with the binomial distribution, N - number of MGI identifiers annotated to any category over the set of all MGI identifiers, M - number of MGI identifiers annotated to the given category, K - number of MGI identifiers tested in the list, and x - number of MGI identifiers in the list annotated to the given category. Annotations for (Q) GO Biological Process (GOBP); (R) GO Molecular Function (GOMF); (S) InterPro; (T) KEGG; and (U) MGI Phenotype.
(0.36 MB XLS)

Table S4 Details of Functional Category Analysis for Tissue Specific Expression (With 2-Fold Change Requirement). Over-representation of annotations was tested for MGI identifiers represented among the set of 1506 probes with at least one prominence-specific difference as determined by limma with fdr = 0.01 and requiring at least a 2-fold change. A prominence-specific subset of the 1506 probes was created from probes showing at least one increase in expression over the other two prominences for the same time point, thereby excluding a probe from a given prominence-specific list if it had the lowest expression in that prominence of the three prominence. For each term, values used in calculating the binomial distribution are given as follows for each of the three prominences (P for mandibular (MdP), X for maxillary (MxP) and N for frontonasal (FNP)): H - whether the category was called significant at the (unadjusted) 0.05 level with the binomial distribution, N - number of MGI identifiers annotated to any category over the set of all MGI identifiers, M - number of MGI identifiers annotated to the given category, K - number of MGI identifiers tested in the list, and x - number of MGI identifiers in the list annotated to the given category. Annotations for (A) GO Biological Process (GOBP); (B) GO Molecular Function (GOMF); (C) InterPro; (D) KEGG; and (E) MGI Phenotype.
(0.26 MB XLS)
