# Supplementary figures and images for: Spatial and Temporal Analysis of Gene Expression during Growth and Fusion of the Mouse Facial Prominences
Source: PLoS One. 2009 Dec 16;4(12):e8066. doi: 10.1371/journal.pone.0008066 (PMC2789411; doi:10.1371/journal.pone.0008066)

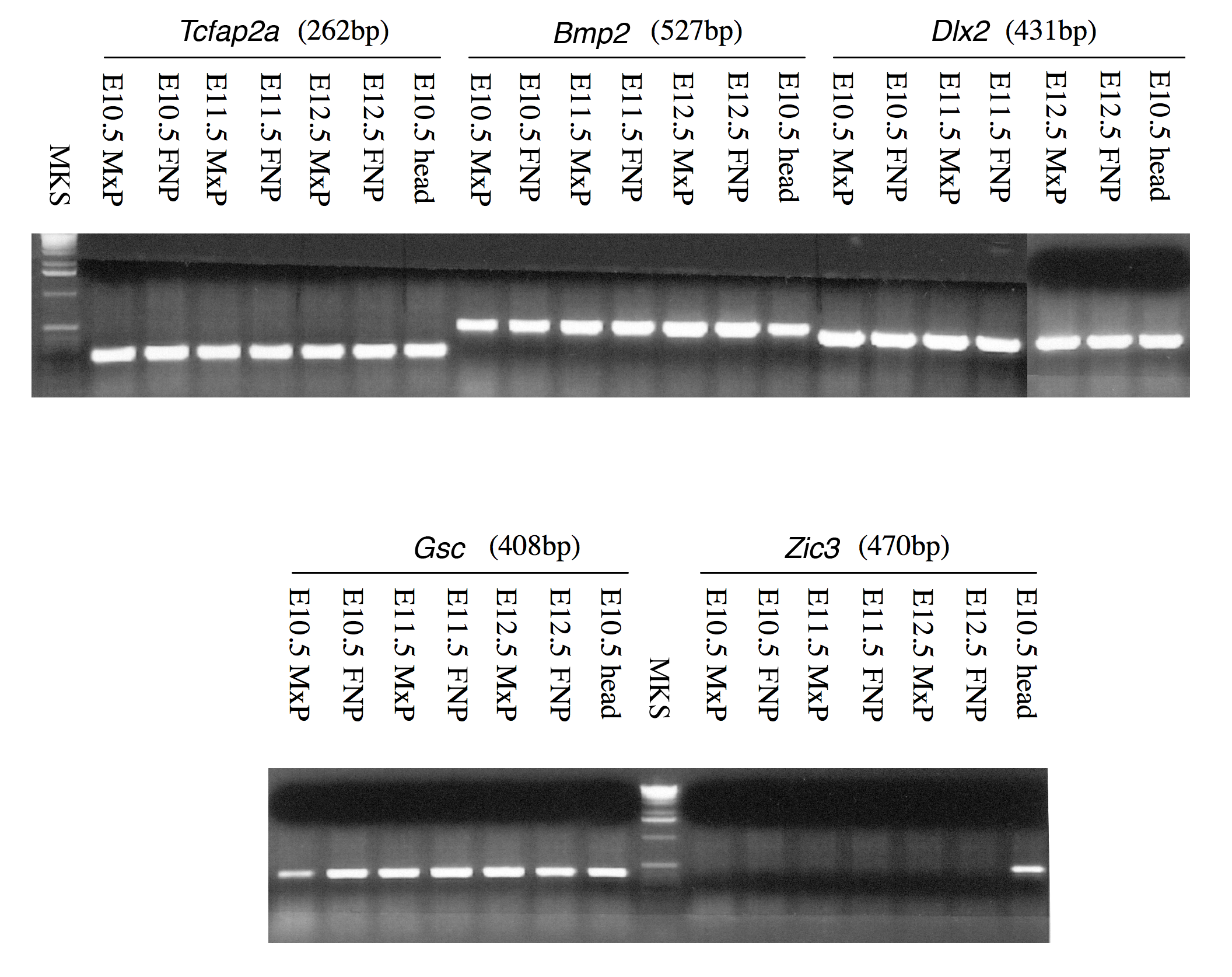

Supplement: Figure S1 — Assessment of RNA quality prior to microarray analysis. A representative RT-PCR analysis on RNA isolated from the FNP and MxP for four genes expected to be expressed in the facial prominences (Tcfap2a, Bmp2, Dlx2, and Gsc). The sizes of the expected products are shown next to the gene name. Zic3 is expressed in forebrain tissue and its absence from the facial prominence RNA samples following RT-PCR and gel electrophoresis was indicative of lack of contamination by CNS tissue. The intact mouse head sample serves as a positive control for all RT-PCR reactions. MKS, DNA ladder size markers. (1.31 MB TIF) [file pone.0008066.s001.tif]

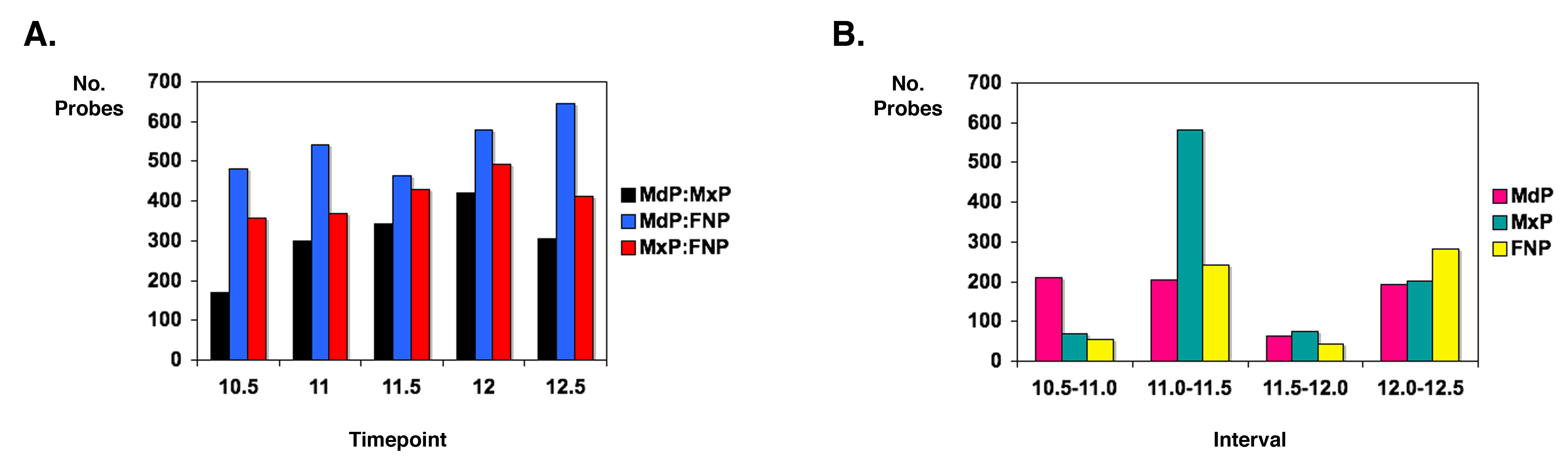

Supplement: Figure S2 — Spatial and Temporal Specific Gene Expression Differences in the Developing Facial Prominences. A. A bar graph illustrating the number of probes showing significant differences between the two prominences indicated at a given time point assessed using limma based on a 1% false discovery rate and a 2-fold expression difference. B. Bar graph showing the number of probe sets showing a significant increase or decrease between adjacent time points within a particular prominence using the same statistical criteria employed in A. (0.56 MB TIF) [file pone.0008066.s002.tif]

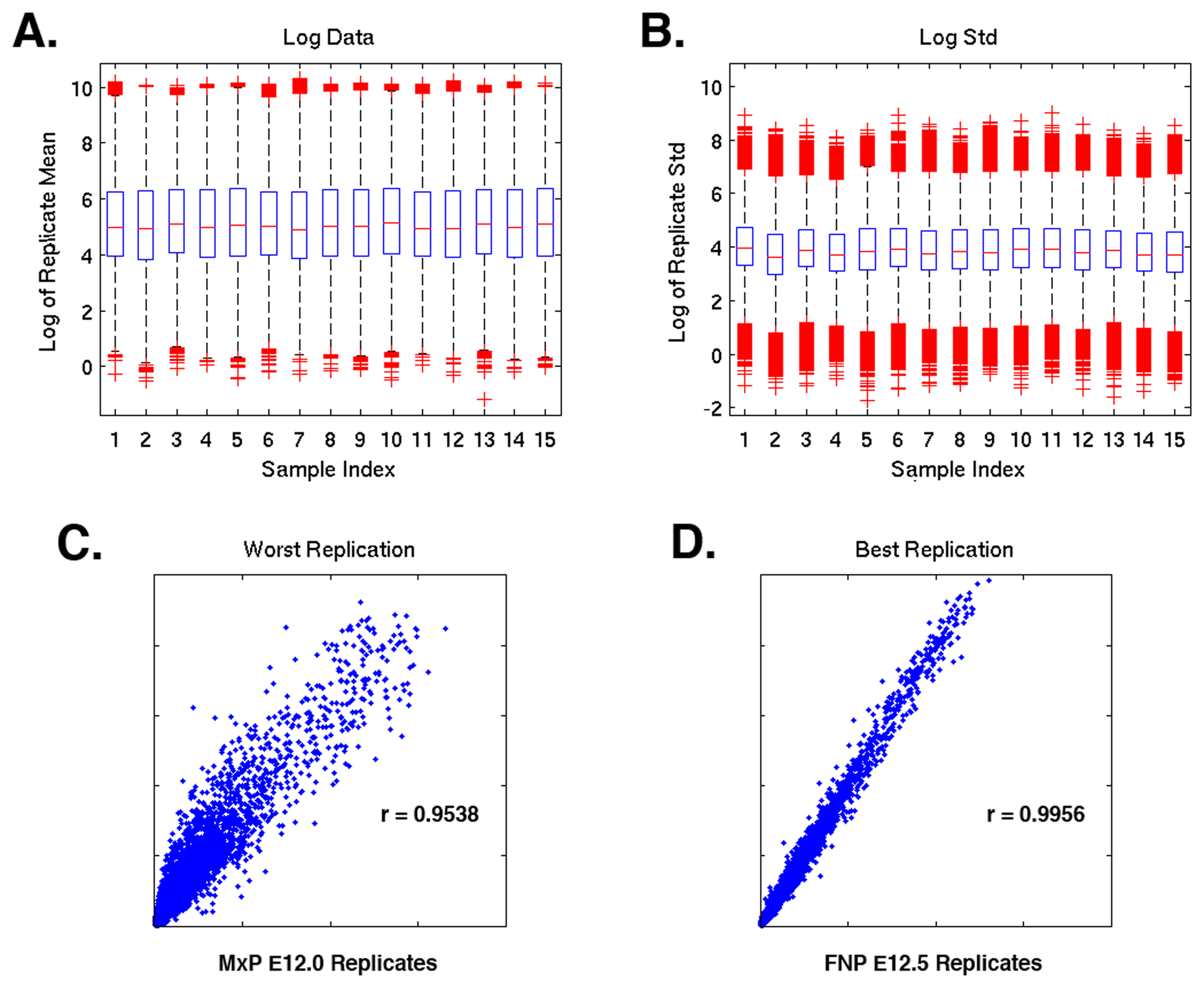

Supplement: Figure S3 — Analysis of Signal values among replicates. A–B. Boxplot of logarithm of mean and standard deviation of the Signal values among seven replicates. The horizontal axis shows the sample index 1–15 corresponding to time points E10.5 to E12.5 in MdP (1–5), MxP (6–10) and FNP (11–15) respectively. Boxes have lines at the lower quartile, median and upper quartile values while whiskers extending from the box show the remaining data range. The consistency of box size and median level across all boxes shows reproducibility among replicates in all prominences and at all time points. C–D. Worst (C) and best (D) replication among a pair of replicates. Each dot represents the Signal value for a probe. The identity of the sample is given along the axes and coefficient r measures linear correlation. The wide and slim spread of points in each plot demonstrates high and low quality replication, respectively. (0.37 MB TIF) [file pone.0008066.s003.tif]

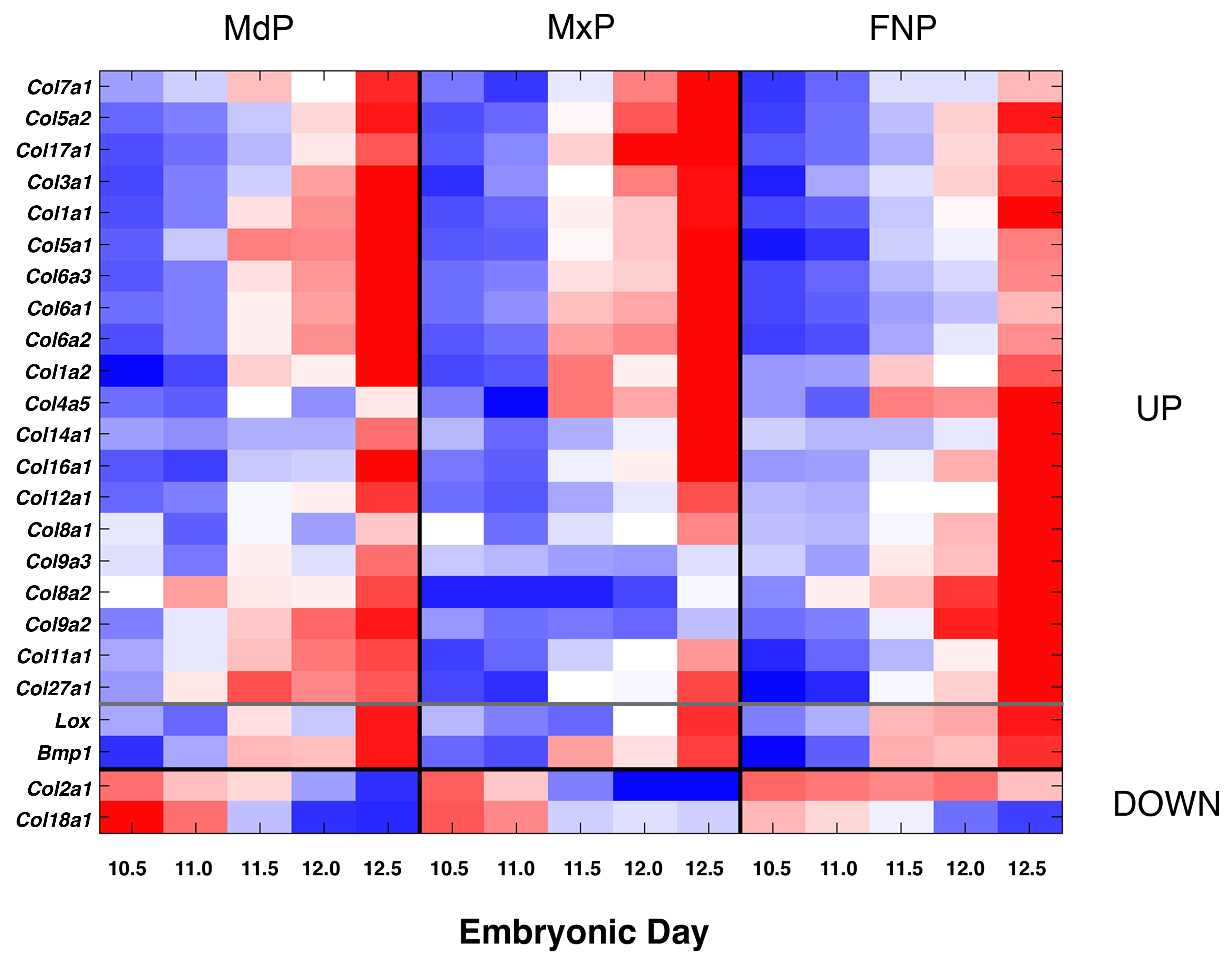

Supplement: Figure S4 — Heatmap of collagen and collagen processing enzyme gene expression. The genes analyzed are shown on the left, the prominences at the top, the time points at the bottom and the class of profile at the right. The grey line marks the separation of up-regulated collagen genes from processing enzymes. Data for a given gene is the average of data for all probe sets representing that gene (first averaged within the seven biological replicates per time point) and then the resulting vector is scaled to have a mean of zero and a magnitude of one. Blue and red indicate low and high expression levels, respectively. (0.87 MB TIF) [file pone.0008066.s004.tif]

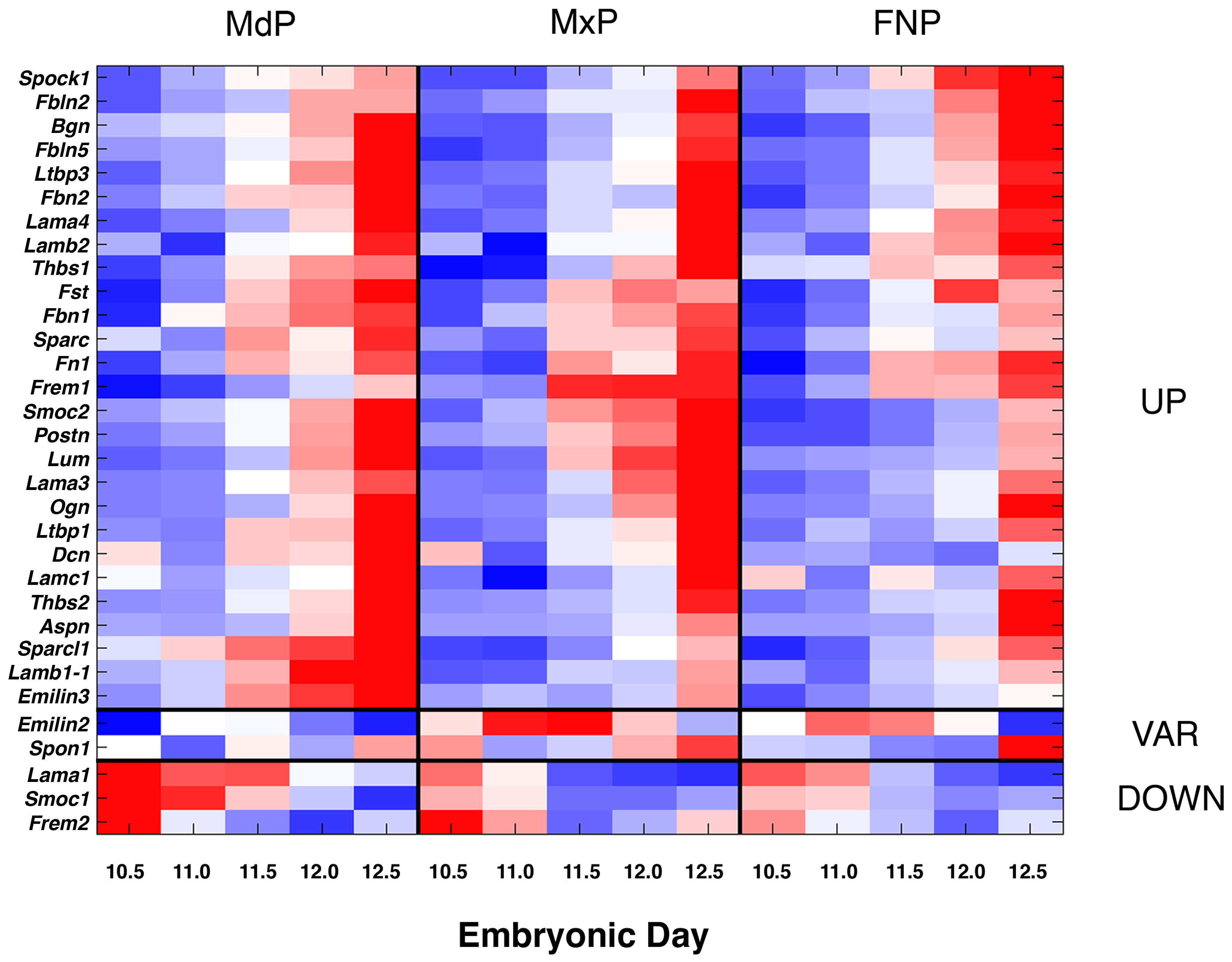

Supplement: Figure S5 — Heatmap of extracellular matrix component gene expression. The genes analyzed are shown on the left, the prominences at the top, the time points at the bottom and the class of profile at the right. Data for a given gene is the average of data for all probe sets representing that gene (first averaged within the seven biological replicates per time point) and then the resulting vector is scaled to have a mean of zero and a magnitude of one. Blue and red indicate low and high expression levels, respectively. VAR, variable profile but still coordinately regulated. (1.04 MB TIF) [file pone.0008066.s005.tif]

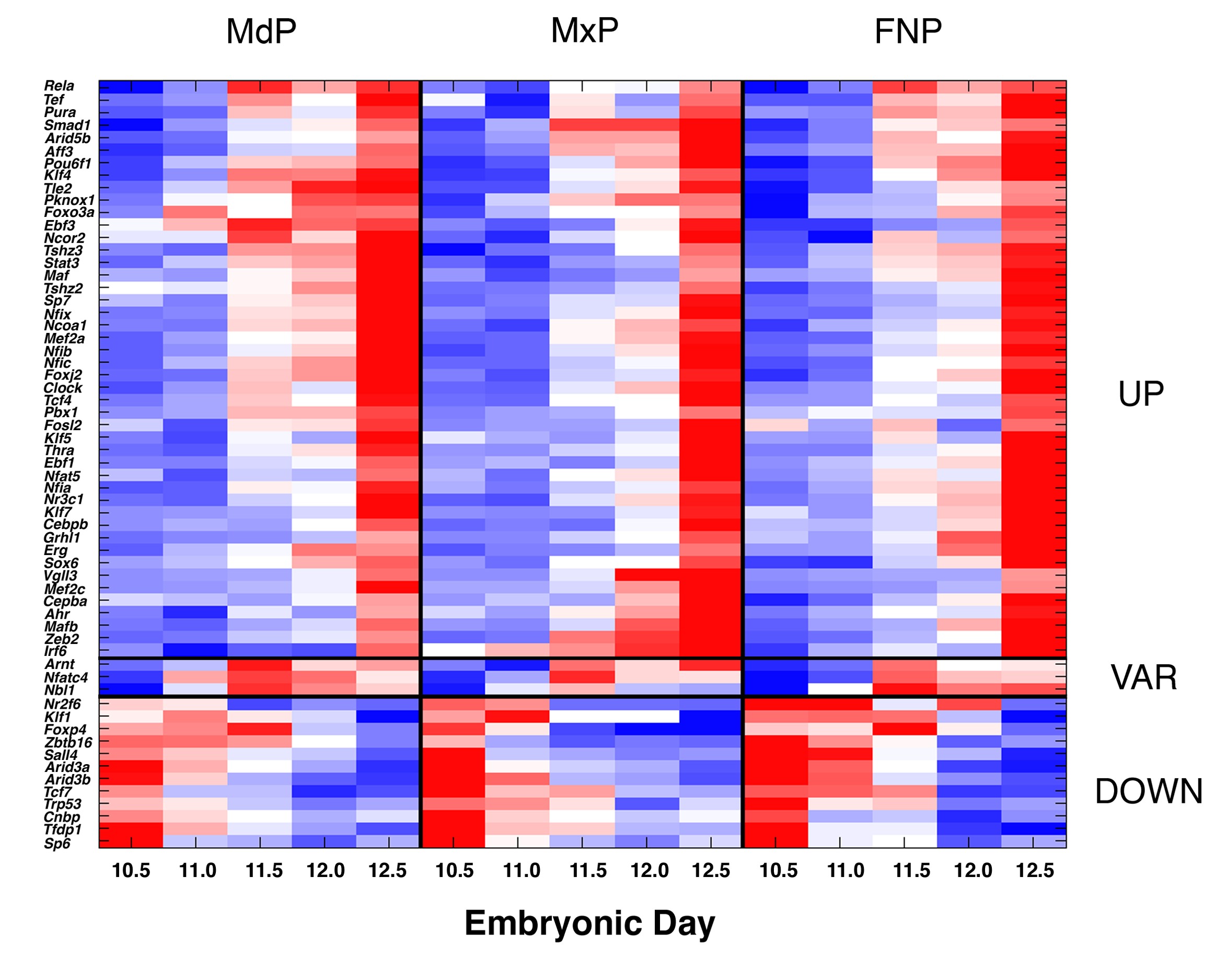

Supplement: Figure S6 — Heatmap of transcription factors coordinately regulated in all prominences. The genes analyzed are shown on the left, the prominences at the top, the time points at the bottom and the class of profile at the right. Data for a given gene is the average of data for all probe sets representing that gene (first averaged within the seven biological replicates per time point) and then the resulting vector is scaled to have a mean of zero and a magnitude of one. Blue and red indicate low and high expression levels, respectively. VAR, variable profile but still coordinately regulated. (0.59 MB TIF) [file pone.0008066.s006.tif]

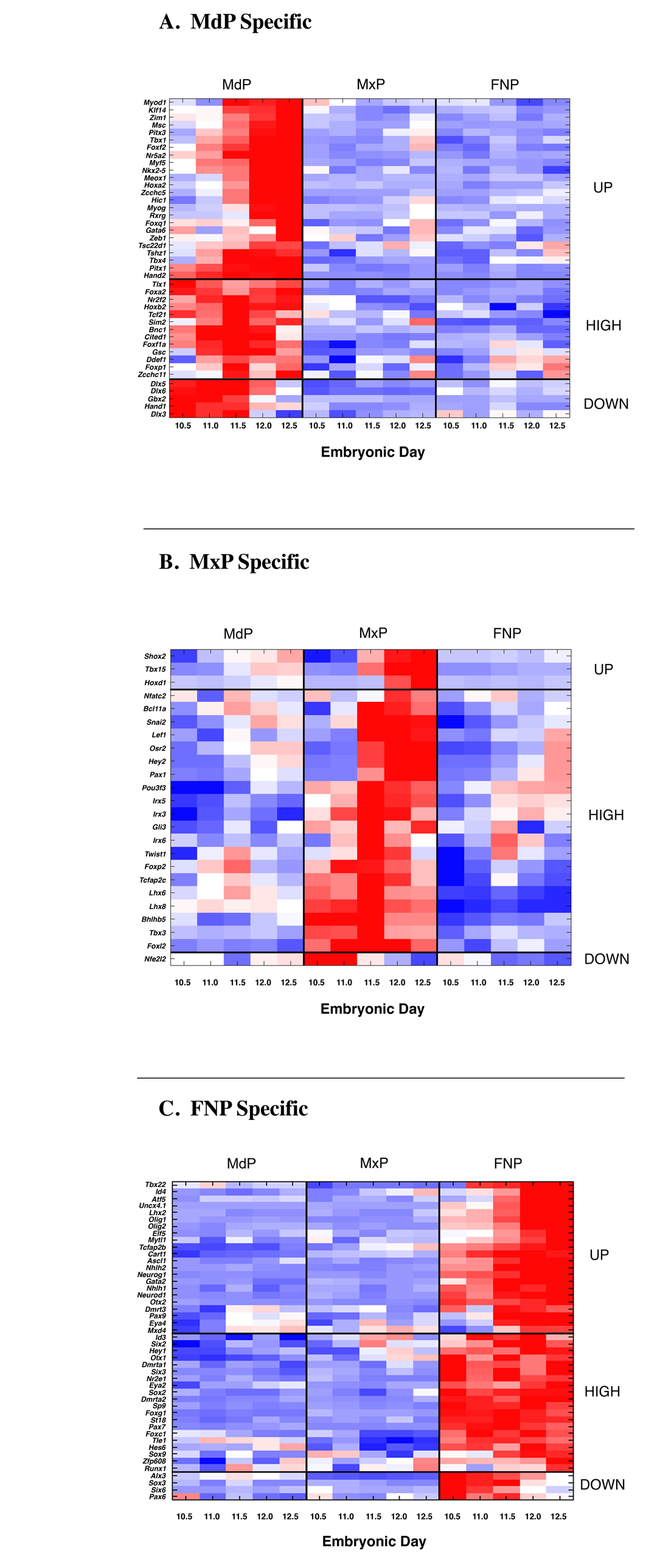

Supplement: Figure S7 — Heatmap of transcription factors highly expressed in a single prominence. The genes analyzed are shown on the left, the prominences at the top, the time points at the bottom and the class of profile at the right. Data for a given gene is the average of data for all probe sets representing that gene (first averaged within the seven biological replicates per time point) and then the resulting vector is scaled to have a mean of zero and a magnitude of one. Blue and red indicate low and high expression levels, respectively. HIGH, expression is generally higher throughout the time course than in the other prominences, although not necessarily increasing or decreasing. (3.14 MB TIF) [file pone.0008066.s007.tif]

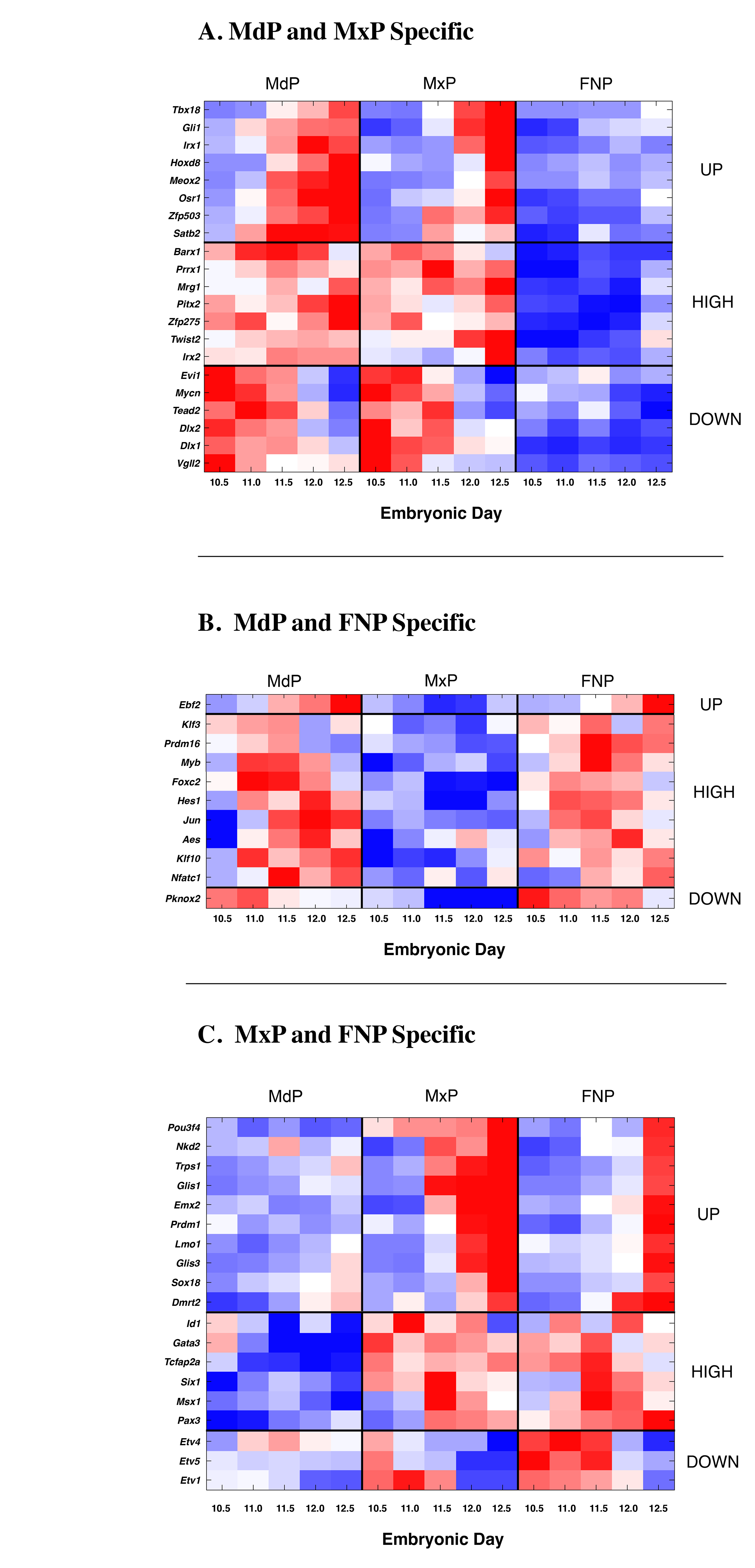

Supplement: Figure S8 — Heatmap of transcription factors highly expressed in two of the three prominences. The genes analyzed are shown on the left, the prominences at the top, the time points at the bottom and the class of profile at the right. Data for a given gene is the average of data for all probe sets representing that gene (first averaged within the seven biological replicates per time point) and then the resulting vector is scaled to have a mean of zero and a magnitude of one. Blue and red indicate low and high expression levels, respectively. HIGH, expression is generally higher throughout the time course in two of the three prominences, although not necessarily increasing or decreasing. (2.68 MB TIF) [file pone.0008066.s008.tif]

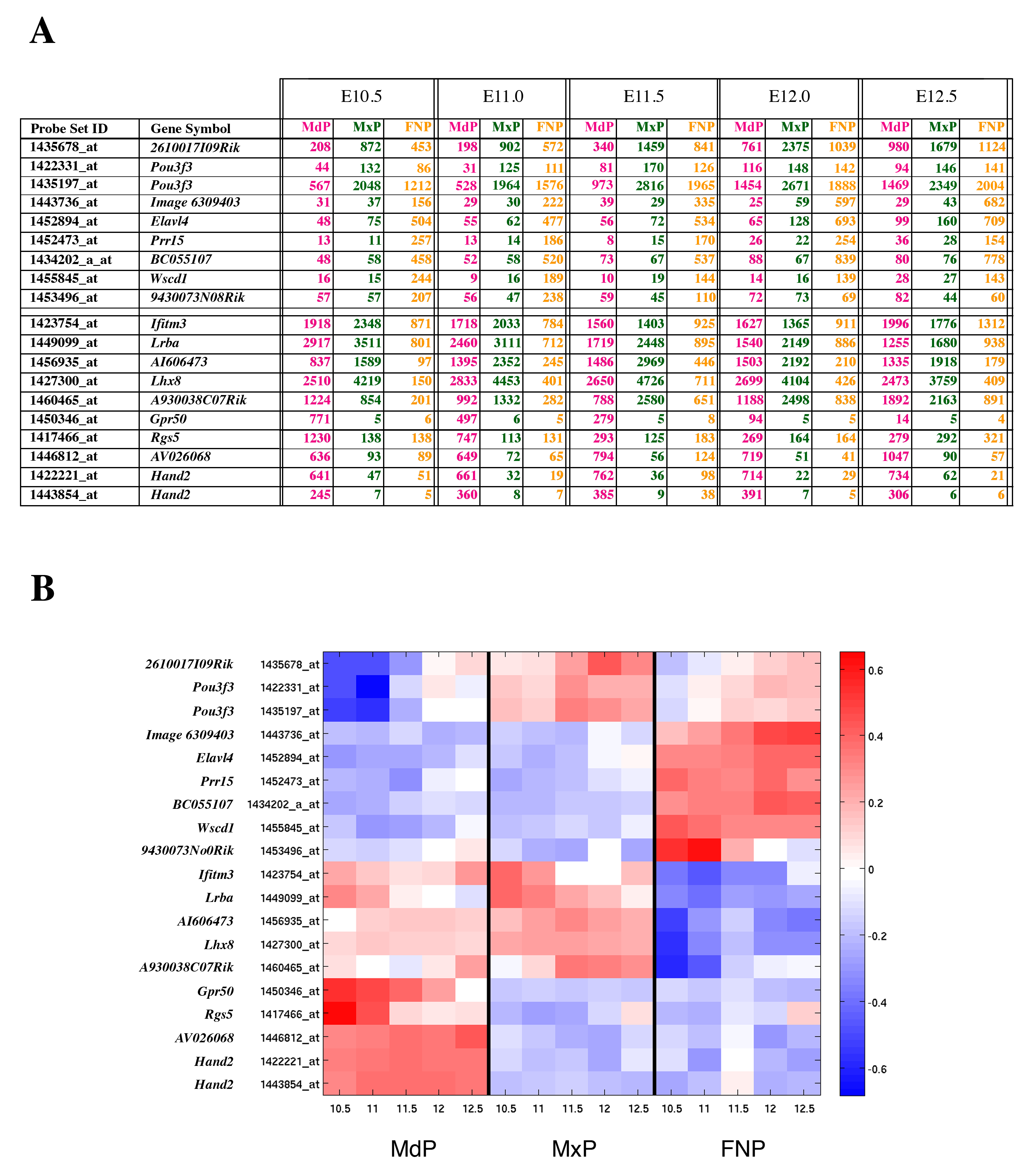

Supplement: Figure S9 — (A). Biological verification of the dataset using the novel genes associated with orofacial expression as well as the previously characterized linked genes. Raw data for the genes and associated probe sets (left) indicated at the five time points in the three prominences. (B). Corresponding heatmap showing the expression data for each probe scaled such that the vector of log2 expression values for a probe (averaged among replicate samples per time point) has a mean of zero and a magnitude of one. Red and blue indicate high and low expression, respectively. (1.16 MB TIF) [file pone.0008066.s009.tif]
