# Supplementary material for: Spatial and Temporal Analysis of Gene Expression during Growth and Fusion of the Mouse Facial Prominences
Source: PLoS One. 2009 Dec 16;4(12):e8066. doi: 10.1371/journal.pone.0008066 (PMC2789411; doi:10.1371/journal.pone.0008066)
Supplement: Figure S10 — Bidirectional transcription units associated with orofacial gene expression. The analysis of novel genes associated with expression in the facial prominences indicated that at least three mouse genes displaying similar expression patterns to known genes were also closely linked to them in the mouse genome and transcribed in the opposite direction. These finding suggest that they share common cis-acting sequences responsible for their similar patterns of gene expression. The distances between the pairs 2610017I09Rik and Pou3f3, AI606473 and Lhx8, and AV026068 and Hand2 are approximately 2.3 kb, 260 bp and 60–220 bp respectively. A bidirectional arrangement of these gene pairs is also conserved in the human genome (human cDNA or gene names are shown in blue), with the following caveats. The AK096498 (Homo sapiens cDNA FLJ39179 fis, clone OCBBF2004147) mRNA initiates ∼4 kb upstream of POU3F3, but other spliced transcripts from this locus begin only ∼2.3 kb upstream. The human RefSeq for LHX8 contains two upstream exons that are not present in the mouse RefSeq. The opposite strand transcript from AK055631 would therefore initiate from within the second intron of LHX8. However, we suspect (based on a protein sequence comparison between mammalian species) that the human RefSeq may have been derived from an atypical transcript and that the arrangement we have noted in the mouse may also hold in the human genome. Information was obtained from NCBI (http://www.ncbi.nlm.nih.gov/) and the UCSC genome browser (http://genome.ucsc.edu/) using the Mouse July 2007 (mm9) Assembly and the Human Feb. 2009 (hg19) Assembly. (0.03 MB DOC) [file pone.0008066.s010.doc]

***Pou3f3***

***2610017I09Rik***

***(AK096498)***

**Mouse chr 1**

***Lhx8***

***AI606473***

***(AK055631)***

**Mouse chr 3**

***Hand2***

***AV026068***

***(Nbla00301)***

**Mouse chr 8**

**100bp**
