# Supplementary material for: Spatial and Temporal Analysis of Gene Expression during Growth and Fusion of the Mouse Facial Prominences
Source: PLoS One. 2009 Dec 16;4(12):e8066. doi: 10.1371/journal.pone.0008066 (PMC2789411; doi:10.1371/journal.pone.0008066)
Supplement: Table S1 — Gene specific PCR primer sequences and PCR product sizes. (0.26 MB DOC) [file pone.0008066.s011.doc]

**Table S1. Gene Specific PCR Primer Sequences and PCR Product Sizes**

| Gene/ID | Forward Primer | Reverse Primer | Size  (bp) |
| --- | --- | --- | --- |
|  |  |  |  |
| *Bmp2* | Bmp2F:  5’-GCTCGAGGCTGTGTGTCAGCACTTGG-3’ | Bmp2R:  5’-GCTGAGCAGCCTCAACTCAAATTCG-3’ | 527 |
| *Dlx2* | Dlx2F:  5’-GCACGCGTCCTGTAGCTAGAGGCTCG-3’ | Dlx2R:  5’-GGTAGGTGATAGGGTGGAGTAGGACC-3’ | 431 |
| *Gsc* | GscF:  5’-GGTTCTGTACTGGTGTCTCCGGTGC-3’ | GscR:  5’-CGTCAGCTGTCCGAGTCCAAATCG-3’ | 408 |
| *Tcfap2a* | ap2exon5up:  5’-AGCGAAGTCTAAGAATGGAGG-3’ | ap2exon6down:  5’-GTGGCCAGGAGCATGTTTTTTCTTGC-3’ | 262 |
| *Zic3* | Zic3F:  5'-TCTAGATTCCTTACAATGTCAGT-3' | Zic3R:  5'-AAGAAGCACTTTAACCATGAG-3' | 470 |
| *1435678_at* | Da4MF3:  5’-CCTGAGTCAACATGGCCGTTC-3’ | Da4MR2:  5’-CAACTTGGAGGAAAAGTCTTGTC-3’ | 434 |
| *1446812_at* | Da6MF:  5’-GCACAGTGTCCTGAGAATCCTGGAC-3’ | Da6MR2:  5’-GGTAACACAAAGACAGACTGCATAG-3’ | 516 |
| *1452894_at*  *(Elavl4)* | Da7F:  5’-CCATGCTGAACCCACTAAGTG-3’ | Da7MR:  5’-CTAGCAAGATTAACATGAAGTC-3’ | 436 |
| *1443736_at* | Da8F  5’-GCACTGCCAAGGTGCCAGACCAG -3’ | Da8R  5’- CCTCAGCCACATAGAACTTGGAGCC-3’ | 566 |
| *1436578_at* | Da9M2F:  5’-GGAGGAATCTTGCTATTGCACGTCAG-3’ | Da9M2R:  5’-CTCTCTGCTCAAGCTGTTGCTGG-3’ | 553 |
| *1438799_at*  *(Dlx6os1)* | Da10MF:  5’-GTTGTCTGACCTCATTGGAAATC-3’ | Da10MR:  5’-CCCTTGATTTTCTAAACTCCTATC-3’ | 444 |
| *1429991_at*  *(Fezf1)* | Da11MF:  5’-CTTTAATGCGCACTATAACTTAACC-3’ | Da11MR:  5’-GCTGCTTGGCTCCCCAGTAGGAGTGC-3’ | 468 |
| *1434202_a_at*  *1434203_at* | Da12F:  5’-GGTTGTCACCTTACAGGTACATG-3’ | Da12R:  5’-GATGGAGAGCTGTTGGCACTTG-3’ | 479 |
| *1437355_at*  *(Zcchc5)* | Da13MF:  5’-GCTACGTTACAGAGGAGAGTTGGAAC-3’ | Da13MR:  5’-GGTTCATGCTCTTTGACTGCTCC-3’ | 530 |
| *1444596_at*  *(Pax7)* | Da14F:  5’-ATGTGTCAAGATACCTGTG-3’ | Da14R:  5’-ACAAGCAGCAGGTAGTTAAAG-3’ | 310 |
| *1417466_at*  *(Rgs5)* | Da21F:  5’-CCTTGAGTAAATTGCCTGAGAAAG-3’ | Da21R:  5’-CCTCATGCATTTGAAATGAAAATGC-3’ | 545 |
| *1431094_at* | Da22F:  5’-CCTAGGTCACACTTTGTGCATATG-3’ | Da22R:  5’-GAGAAGTAACAGAAGGTGCAGAATTC-3’ | 581 |
| *1436361_at (Vgll2)* | Da23F:  5’-GGTCTCAGCGTGGACTCAGGTAAGC-3’ | Da23R:  5’-CCAATATAACCAATAGGCAACC-3’ | 517 |
| *1436361_at (Vgll2*) | Da23MF:  5’-GGAGCCATGAGCTGTCTGGATGTTATG-3’ | Da23Mr:  5’-GGAGAGTAGGGGTCGGTGGCAAAGG-3’ | 539 |
| *1450346_at (Gpr50)* | Da24F:  5’-CCTGTCAATGTGCTCACTGTGTTG-3’ | Da24R:  5’-GGACAGAGGAGGCTGGCTTAGG-3’ | 520 |
| *1452473_at*  *(Prr15)* | Da25F:  5’-CCAGGTTGTTCCGACAGTGTGC-3’ | Da25R:  5’-CGACTATTGCCTGTTTTCTCCTCAC-3’ | 568 |
| *1453496_at* | Da26F:  5’-GGACTCCAGTGTAAGATGCTCTGG-3’ | Da26R:  5’-CGTCTGAGTGAGTACACTGACAC-3’ | 528 |
| *1455845_at*  *(Wscd1)* | Da27F:  5’-GCTGCTGGTCGTGCACTATGAGG-3’ | Da27R:  5’-CAAGTGGCTTAGGCACATTAAAATGG-3’ | 567 |
| *1456440_s_at*  *(St8sia6)* | Da28F:  5’-CACAGCAATTAGGAGCAACTTG-3’ | Da28R:  5’-GGAGAAATTTGACACGTGTTGG-3’ | 523 |
| *1456521_at*  *(Nr5a2)* | Da29F:  5’-GCAGAGGAGCCACAGTCCAAAGACTCG-3’ | Da29R:  5’-GTCATCAGTCAAAGCTCAGATCCAAAGC-3’ | 556 |
| *1456935_at* | Da30F:  5’-CCTCTCTGGAGTTGCGATCCTCCAGAGG-3’ | Da30R:  5’-GGTGTTCAGGCATCCACCTTCTTGG-3’ | 590 |
| *1460465_at* | Da31F:  5’-CGATTCATCTGTGATTCCAGATGG-3’ | Da31R:  5’-CCACCATGAAGGCATCATCTGCATTC-3’ | 596 |
| *1423754_at*  *(Ifitm3)* | Da41F:  5’-GCTCCATCCTTTGCCCTTCAGTGCTG-3’ | Da41R:  5’-GCAGCTGCGGAGGGTGAAGCACTTCAG-3’ | 504 |
| *1449099_at*  *(Lrba)* | Da44F:  5’-GGATACAGACAGTGGAACCAATAATCC-3’ | Da44R:  5’-GGTGCTGCTTACACAGTACCTGGTCG-3’ | 571 |
